# Wound Healing Composite Materials of Bacterial Cellulose and Zinc Oxide Nanoparticles with Immobilized Betulin Diphosphate

**DOI:** 10.3390/nano11030713

**Published:** 2021-03-12

**Authors:** Nina Melnikova, Alexander Knyazev, Viktor Nikolskiy, Peter Peretyagin, Kseniia Belyaeva, Natalia Nazarova, Elena Liyaskina, Darina Malygina, Viktor Revin

**Affiliations:** 1 Faculty of Chemistry, Lobachevsky University, 23/5 Gagarin Av., 603950 Nizhny Novgorod, Russia; knyazevav@gmail.com; 2 Nizhni Novgorod Regional Clinical Hospital named after N.A. Semashko, 190 Rodionova str., 603126 Nizhny Novgorod, Russia; viktor22031@yandex.ru; 3 Department of Experimental Medicine, Privolzhsky Research Medical University, 10/1 Minin sq., 603950 Nizhny Novgorod, Russia; peretyaginpv@gmail.com (P.P.); skoln94@mail.ru (K.B.); 4 Department of Biotechnology, Bioengineering and Biochemistry, National Research Ogarev Mordovia State University, 68 Bolshevistskaya str., 430005 Saransk, Russia; fac-bio@adm.mrsu.ru (N.N.); liyaskina@yandex.ru (E.L.); revinvv2010@yandex.ru (V.R.); 5 Department of Pharmaceutical Chemistry, Privolzhsky Research Medical University, 10/1 Minin sq., 603950 Nizhny Novgorod, Russia; mds73@yandex.ru

**Keywords:** bacterial cellulose, wound dressings, zinc oxide nanoparticles, betulin diphosphate, antioxidant properties, burns

## Abstract

A design of new nanocomposites of bacterial cellulose (BC) and betulin diphosphate (BDP) pre-impregnated into the surface of zinc oxide nanoparticles (ZnO NPs) for the production of wound dressings is proposed. The sizes of crystalline BC and ZnO NPs (5–25%) corresponded to 5–6 nm and 10–18 nm, respectively (powder X-ray diffractometry (PXRD), Fourier-infrared (FTIR), ultraviolet (UV), atomic absorption (AAS) and photoluminescence (PL) spectroscopies). The biological activity of the wound dressings “BC-ZnO NPs-BDP” was investigated in rats using a burn wound model. Morpho-histological studies have shown that more intensive healing was observed during treatment with hydrophilic nanocomposites than the oleophilic standard (ZnO NPs-BDP oleogel; *p* < 0.001). Treatment by both hydrophilic and lipophilic agents led to increases in antioxidant enzyme activity (superoxide dismutase (SOD), catalase) in erythrocytes and decreases in the malondialdehyde (MDA) concentration by 7, 10 and 21 days (*p* < 0.001). The microcirculation index was restored on the 3rd day after burn under treatment with BC-ZnO NPs-BDP wound dressings. The results of effective wound healing with BC-ZnO NPs-BDP nanocomposites can be explained by the synergistic effect of all nanocomposite components, which regulate oxygenation and microcirculation, reducing hypoxia and oxidative stress in a burn wound.

## 1. Introduction

Recently, biodegradable materials, biocompatible to body tissues, based on bacterial cellulose (BC) for the treatment of skin diseases, burns and wounds of various etiologies, have attracted great interest [1]. The need for these materials is due to their bactericidal effect, their ability to absorb large amounts of exudate and remove unwanted products from the wound, and their possibility of immobilizing active pharmaceutical ingredients (antibiotics, steroid hormones, etc.) into the bacterial cellulose matrix. In the treatment of burns, biologically active substances that promote collagen production, such as pentacyclic triterpenoids, including betulinic acid derivatives [2], are of great importance. In rats and mice experiments, we have previously shown that betulin diphosphate (BDP) exhibits antitumor, antioxidant, and anti-burn properties [3,4].

The components for the formation of BC nanomaterials can improve its properties and act as a vector for delivering drugs with low bioavailability. It is a convenient method for obtaining new wound dressings and drugs.

The most acceptable form of new wound dressings is BC nanomaterials with nanoscale inorganic particles, such as gold, silver, and metal oxides (copper oxide, titanium oxide, zinc oxide, etc.). Among the BC nanomaterials with metal nanoparticles, composites with zinc oxide nanoparticles (ZnO NPs), which are of particular importance in treating skin diseases should be noted [5,6,7,8,9,10,11,12,13,14,15]. Several factors determine the effect of zinc oxide on wound healing: (i) zinc ions are biogenic; (ii) zinc ions are part of many enzymes and indirectly control the activity of more than 200 enzymes; (iii) they exhibit immunomodulatory properties [16]; and (iv) a small part of zinc ions produced by ZnO NPs have high wound skin permeability [17,18].

Zinc oxide nanoparticles can affect indirect antioxidant properties and be potential regulators of redox imbalance during the burn disease due to tissue hypoxia in the wound. ZnO NPs’ influence on oxidative stress, increases the generation of reactive oxygen species (ROS) but increases antioxidant enzyme defense and regulates energy metabolism [18,19,20,21,22].

The destruction of tissue and damage to the epithelium leads to a disruption in the microcirculation system, increasing hypoxia and suppressing tissue respiration, which slows down the repair process [23,24]. Zinc oxide nanoparticles promote the regeneration of damaged tissues by activating collagen synthesis [16,25,26].

A serious obstacle to the widespread medical use of ZnO NPs is the possibility of forming rigid protein crowns (or protein composites) on the surface of nanoparticles in a biological environment. The formation of protein corona (PC) most rapidly occurs on the surface of hydrophilic unprotected particles in a liquid medium [27,28,29,30,31]. The presence or absence of the PC usually affects the cytotoxicity of the material. Traditional protection of ZnO NPs is functionalization with protein repellent molecules such as (Polyethylene glycol) PEG or phosphoric polyesters [32,33], which reduce protein adsorption and decrease binding to surface proteins.

In previous work [34], we studied adsorption on the surface of ZnO NPs, Langmuir monolayers of BDP on an aqueous subphase containing zinc ions. We transferred BDP monolayers onto quartz and CaF_2_ using the FTIR method. We proved that zinc ions are strongly bound on the surface of ZnO NPs with polar BDP phosphate groups, providing hydrophobicity of modified ZnO NPs. We can assume that the protection of ZnO NPs by BDP and pretreatment of BC with BDP can reduce the protein crown’s formation.

BDP, which has anti-burn properties and has two active phosphate groups in its molecule, performs several functions in a nanocomposite. BDP can interact with zinc ions of ZnO NPs, protect the nanoparticles from aggregation, and be sorbed into BC.

In this work, we developed new nanocomposites of BC with ZnO NPs and betulin diphosphate pre-impregnated into the surface of ZnO NPs or BC for wound dressings and the study of their biological activity on a thermal burn model in rats.

The objectives of the study were: (i) estimation of the physicochemical properties of “protected ZnO NPs”, “BDP-impregnated ZnO NPs”, and BC-ZnO NPs-BDP nanocomposites; (ii) zinc ions release and cell viability analysis; (iii) investigation of wound healing properties of the obtained BC-ZnO NPs-BDP nanocomposites in an experiment in rats on a model of deep second-degree burn wound according to morphological and histological studies and microcirculation data; and (iv) estimation of biochemical indexes (the activity of enzymatic antioxidant defense (superoxide dismutase (SOD), catalase), malondialdehyde (MDA) level) during the treatment with the BC-ZnO NPs-BDP nanocomposite.

## 2. Materials and Methods

### 2.1. Preparation of BC

BC was produced in a static culture medium by *Komagataeibacter sucrofermentans* H-110, which was isolated from Kombucha tea and identified by sequencing the amplified product of 16S rRNA [35]. A strain was deposited in the Russian National Collection of Industrial Microorganisms (VKPM) (Accession No. VKPM: B-11267). For the production of BC, a Hestrin and Schramm (HS) medium that contained glucose (20 g∙L^−1^), peptone (5 g∙L^−1^), yeast extract (5 g∙L^−1^), citric acid (1.15 g∙L^−1^), and disodium hydrogen phosphate (2.7 g∙L^−1^) at a pH of 6.0 was used. The culture medium was autoclaved for 20 min at 120 °C. Further, the medium was inoculated with 10% (*v*/*v*) inoculum. To prepare the inoculum, *K. sucrofermentans* H-110 was transferred aseptically from an agar plate to a 250 mL Erlenmeyer flask containing 100 mL of culture medium and placed into a shaker incubator (model ES-20/60, BIOSAN, Latvia) at 28 °C for 24 h at 250 rpm. The BC was produced in static conditions at 28 °C for 5 d. After the incubation, the BC was collected, washed thoroughly with de-ionized water to remove medium components, and treated with 1% (*w*/*v*) sodium hydroxide solution at 80 °C for 1 h to eliminate the bacterial cells. Further, the BC was rinsed extensively with 6% (*v*/*v*) acetic acid and then de-ionized water until the pH became neutral. If necessary, the purified BC was dried to a constant weight at 60 °C. The ^13^C nuclear magnetic resonance (NMR) spectrum of BC used in this study is typical spectrum of BC crystal structure [36,37,38,39] (Figure S1). There are four spectral regions in the BC spectrum: (i) C-1 acetal signal (105.57 ppm); (ii) C-4 signals (double peak corresponds to the crystalline (89.4 ppm, approximately 85%) and amorphous (84.47 ppm, approximately 15%) forms of BC); (iii) C-2, C-3, and C-5 signals in the pyranose ring in the region of 71.47–75.04 ppm; and (iv) C-6 (65.76 ppm).

### 2.2. Betulin-3,28-diphosphate

Betulin-3,28-diphosphate (BDP, 3β,28-diphosphate-lup-20(29)-ene) was synthesized according to the procedure [40].

### 2.3. Zinc Oxide Nanoparticles

Two methods of ZnO NPs preparation were used in this work.

(1) Sol-gel methods using fresh prepared ethanol solutions: 2% NaOH (10 mL), 1.5% zinc acetate dihydrate (30 mL) at 70 °C. NaOH solution was added by drops to zinc acetate dihydrate solution at cooling and mixing in an ice bath for 5–10 min. The precipitation of ZnO NPs from the transparent sol was carried out by addition of n-heptane followed by vigorous stirring. The ratio of volume of n-heptane to sol was 3:2. After centrifugation, the precipitate was collected and redispersed into ethanol. The removal of excess acetate and sodium ions from the dispersed ZnO was accomplished by repetitive washing of ZnO with ethanol and n-heptane [41].

We protected the obtained ZnO NPs before forming the composite by additional treatment with a 0.5 M alcohol solution of BDP at pH 3.75–4.10. In the text, the designation of these nanoparticles is ZnO NPs.

(2) We used freshly prepared ethanol solutions of the 0.025 M zinc acetate dihydrate and a 0.175 M lithium hydroxide solution, which was sonicated for 30 min. Then 1 g of PEG 400 was added to the solution; the solution was re-sonicated for 40 min; after that, we added the alkali solution. The resulting solution was sonicated for 5 min at t = 30 °C. To precipitate the white zinc oxide particles formed in the volume, 5 g of cetyl alcohol was used and stirred for 5–7 min. After separation of the liquid and solid phases, the white precipitate was repeatedly washed with 96% ethanol to remove unreacted substances. The precipitate was dried at room temperature for 3 h, then placed in a desiccator [42].

We protected the obtained ZnO NPs before forming the composite by additional treatment with a 0.5 M alcohol solution of BDP at pH 3.75–4.10. In the text, the designation of these nanoparticles is ZnO NPs-PEG.

UV and PL spectra are shown in Figure S2.

### 2.4. Oleogel ZnO NPs-BDP

Oleogel ZnO NPs-BDP preparation and properties were described in detail in paper [3]. Formulation of oleogel, %: ZnO NPs (5.0), BDP (1.0), and sunflower oil up to 100.0.

### 2.5. Preparation of BC-ZnO NPs-BDP Composites and Their Properties

The BC film was placed in aqueous alcohol solutions containing trisamine (1%), or DDS-Na (0.01%) or benzalkonium chloride (0.1%), and BDP (1%) for swelling for 30 min. After removing the film, a suspension of an aqueous-alcoholic solution of zinc oxide nanoparticles was sprayed onto the surface of an elastic polymer mesh at the rate of 5% ZnO NPs per 100% of the initial BC film. After removing the excess solvent by vacuum drying, the films were closed on both sides with parchment and sealed in cellophane envelopes.

When treating rats with BC-ZnO NPs, BC-BDP, and BC-ZnO NPs-BDP composite coatings, the composite coatings were additionally moistened with 0.9% sodium chloride solution in the presence of 0.01% lidocaine and 0.01% benzalkonium chloride.

X-ray phase analysis was performed on additionally dried samples, which were stored in a desiccator.

An alternative method for the preparation of BC-ZnO NPs composites included the primary treatment of the film with an aqueous solution of Zn(NO_3_)_2_∙4 H_2_O. The BC film was removed and transferred into a shallow container with 0.1 M solution of NaOH for 10 min at 50 °C. Then the film was rinsed with distilled water until the composite pH became neutral, and after that it was dried.

### 2.6. FTIR Analysis

FTIR spectra in 400–4000 cm^−1^ range were measured by an IR Prestige-21 FTIR spectrometer (Shimadzu, Kyoto, Japan) equipped with a KBr beam splitter. A pellet from a well-dried KBr was prepared according to standard cold pressing. The resolution was 0.5 cm^−1^ and the number of scans was 45.

### 2.7. UV Analysis

UV spectra were recorded by UV-1800 (Shimadzu, Kyoto, Japan).

### 2.8. RP-HPLC Analysis

Reversed phase high-performance liquid chromatography analysis was carried out on an LC-20Avp (Shimadzu, Kyoto, Japan) with UV-detection, the column is Discovery C18 (25 cm × 4.6 mm, 5 μm, Supelco).

### 2.9. Powder X-ray Diffraction Analysis

Powder X-ray diffraction patterns were obtained using Shimadzu X-ray diffractometer XRD-6000 (Shimadzu, Kyoto, Japan) at 295(2) K with Cu Kα radiation (λ = 1.5418 Å), in the Bragg–Brentano reflection geometry. The samples were collected in the 2θ range between 5 and 50° with steps of 0.026° and 100 s step size, using a scan speed (°/s) of 0.067335. On the X-ray diffraction patterns of amorphous samples, there are diffraction peaks at 37.5° and 44.0° referring to the cuvette material.

### 2.10. Photoluminescence Analysis

Fluorescent spectra were obtained using spectrofluorimeter RF-600 (Shimadzu, Kyoto, Japan) at the length of 320 nm in the field 350–800 nm in a 10 mm thick cuvette.

### 2.11. Elemental Analysis

Elemental analysis was carried out using atomic absorption spectrophotometer AA-7000 (Shimadzu, Kyoto, Japan) at 213.9 nm. The samples were digested with HNO3:HClO4 (6:1) to leach out zinc metal.

### 2.12. ^13^C Nuclear Magnetic Resonance (NMR) Spectroscopy

The measurements of solid-state ^13^C NMR spectra were performed on a JEOL JNM-ECX400 spectrometer (JEOL, Tokyo, Japan) (9.39 T, 100.5 MHz) in the solid phase at room temperature using a cross-polarization technique (CPMAS) with a rotation speed of 10 kHz in 7 mm zirconium dioxide rotors. The angle of rotation of the sample (VUS) was defined at a rotation speed of 10 kHz. All MAS experiments were carried out at room temperature. Proton decoupling was performed by double-pulse phase modulation (TPPM). The measurements of ^13^C magic angle spinning (MAS) NMR spectra were performed using the rotor synchronization of the echo sequence (RSE) or the one-setting pulse (SP) at a Larmor frequency of 100.6 MHz. To optimize the process of spectrum detection, the relaxation time of carbon nuclei was identified. The impulse duration for an angle of 90° was 6 ms and for 180° was 12 ms, with a total of 256 scans. Spectra were processed using ACD/NMR Processor Academic Edition, version 12.01.

### 2.13. SEM Analysis

The morphology of the samples was obtained by scanning electron microscopy (SEM) on JSM-IT300LV (JEOL, Tokyo, Japan) with the electron probe diameter of about 5 nm and probe current below 0.5 nA (operating voltage 20 kV). The study of the sample surface topography was performed using the low-energy secondary electrons and backscattered electrons under low vacuum mode to eliminate charging.

### 2.14. Specific Surface Areas Analysis

Specific surface areas (SSAs) of the ZnO NPs were analyzed using an analyzer of specific surface area and adsorption porosity ASAP 2020 (Micromeritics, Norcross, GA, USA).

### 2.15. Chemical Composition of BC

Chemical composition of BC was analyzed by determining C/O/N using a Flash EA 1112 CN analyzer (NEOLAB Ltd., Italy). Analysis for major and trace elements was performed with an atomic absorption spectrophotometer AA-7000 (Shimadzu, Kyoto, Japan) after wet mineralization of cellulose samples with a mixture of perchloric and nitric acids.

### 2.16. Surface Charge Measurements

Surface charge of the nanoparticles was measured using the zeta potential mode of the Zetasizer Nano ZSP (Grovewood Road, Malvern, Worcestershire, UK). Suspensions having a solid loading of 0.02 g/L were prepared in the presence (1 × 10^−3^ M) as well as in the absence of BDP water-ethanol (1:1) solution and were allowed to equilibrate for 24 h to reach the steady-state. Surface charge measurements were obtained for samples of sizes 10–20 nm.

### 2.17. Biological Activity

Male Wistar rats (150–200 g) were involved in the study. The animals were purchased from the Animal Breeding Facilities “Andreevka” Federal State Budgetary Institution of Science “Scientific Center for Biomedical Technologies” of the Federal Medical and Biological Agency (Andreevka, Moscow region, Russia). The animals were handled humanely, kept in plastic suspended cages, and placed in a well-ventilated and hygienic rat house under suitable conditions of room temperature (27 ± 2 °C) and humidity. They were given food and water ad libitum and subjected to a natural photoperiod cycle of 12 h light and 12 h dark. The animals were allowed two weeks of acclimatization before the commencement of all animal model experiments in the study.

All blood taking and withdrawal of animals for the experiment were performed under anesthesia, all efforts being made to minimize suffering.

#### 2.17.1. Modeling of Thermal Burns in Animals

The surface of the animal’s back was burned using electromagnetic radiation from an infrared soldering station YaXunXY865D following the requirements of Good Laboratory Practice for experimental modeling of thermal burns in laboratory animals. We used the regime that causes deep second-degree thermal burns. The distance of the infrared heater from the animal’s skin was 15 mm, the temperature on the skin in the heating zone was 60 °C, the heating duration was 23 s, and the power was 100 W. Under these conditions, infrared radiation penetrates to a depth of 3–5 mm [43,44]. Standard thermal burns had an area of 14.0 ± 0.5 cm^2^. The body surface area of each animal was evaluated by the weight of the animal using the Mee–Rubner formula [45]. The minimum weight of the rats was 240 g, the maximum was 330 g, and the average weight of the rats was 285.0 ± 5.0 g. The minimum body surface area of the experimental animals was 436.0 cm^2^, the maximum was 555.0 cm^2^, and the average was 494.0 cm^2^.

#### 2.17.2. Wound Area Measurement

The wound area was measured using a planimetry system. A 2-layered transparent film was placed on the wound, and the outline traced onto the film using a permanent marker. The layer of the film in contact with the wound was discarded. The top layer containing the tracing was retraced onto graph paper; the wound area was calculated.

After measurement, the wound contraction was expressed as the percentage change in the original wound area using the following Formula (1) [46]:

(1) $$\mathrm{Wound}\;\mathrm{contraction}\;\;\frac{\left(\mathrm{Specific}\;\mathrm{day}\;\mathrm{wound}\;\mathrm{area}\right)\cdot 100\%\;}{\left(\mathrm{Original}\;\mathrm{wound}\;\mathrm{area}\right)}$$

#### 2.17.3. Morpho-histology Research

Morpho-histology research of excise samples of wound tissues was made on samples fixed in 10% solution of neutral formalin. Then, the test material was washed in running water and dehydrated and serial processing in ethanol solutions with the increase in the concentration: 50%, 60%, 70%, 80%, 90%, 96%, and 100% for 40 min in each solution. Dehydrated samples were initially soaked in xylene for 30 min and then in paraffin within 2 h. In the final stage, the samples were poured with molted paraffin. A sledge microtome (Chuvash State University named after I.N. Ulyanova, Russia) was used for getting paraffin block slices. The samples were colored by hematoxylin and eosin.

#### 2.17.4. Microcirculation Research

Microcirculation was assessed quantitatively using the LAKK-02 (LASMA, Moscow, Russia). This device transmits continuous wave laser light (30 mW, 890 nm) and white light (20 W, 500–900 nm) to skin tissue near the wound, where it is scattered and collected on the skin surface with fibers of the probe. The movement of erythrocytes causes a Doppler shift, which in turn is detected by the laser light and analyzed by the LAKK-02, that is then computed and displayed as the blood flow velocity. The detected laser signal correlates with the number of moving erythrocytes in tissue and blood flow velocity for calculation microcirculation parameters, using such arbitrary (relative) units (arb. un.) as perfusion units (perf. un.). The rate of microcirculation (the microcirculation level), the regulatory activity of its components, and the degree of shunt path participation with an allowance for the frequency range intervals of the blood flow oscillations in the rats’ microvessels were investigated [47,48].

#### 2.17.5. Biological Analysis In Vitro

In vitro biological analysis was performed using blood stabilized with sodium citrate. Erythrocytes were washed twice with 0.9% NaCl by centrifugation for 10 min at 1600× *g*. The intensity of lipid peroxidation (LPO) was estimated by MDA level in plasma and erythrocytes following the methods by Uchiyama and Mihara [49]. Superoxide dismutase activity (EC 1.15.1.1) was measured in erythrocytes using inhibition of adrenaline auto-oxidation [50]. Catalase activity (EC 1.11.1.6) was determined by spectrophotometry based on the decomposition of hydrogen peroxide by the catalase [51]. The specific activity of the enzymes was calculated from the protein concentration analyzed by the modified Lowry method [52].

#### 2.17.6. Cytotoxicity Tests

We used mouse fibroblast cell culture L929 (tissue culture collections of D.I. Ivanovsky Institute of Virology, Russia). The cells were cultured in Dulbecco’s modified Eagle’s medium (DMEM) (PanEko, Moscow, Russia) with 10% fetal bovine serum (FBS bovine serum (FBS) (HyClone, Pittsburgh, PA, USA) and antibiotics (gentamycin). Conditions: 5% CO_2_, t = 37 °C, and 5% humidity in an MCO-170M incubator (Sanyo, Japan). Cells in the exponential growth phase were dispersed in a 96-well plate (5 × 10^3^ cells/well) for 24 h. Then, we added dispersions of studied wound dressings (BC-BDP, BC-ZnO NPs-BDP) or BDP into the fresh replaced medium for 24 h incubation at 37 °C. The control sample was the wells containing only the cells. The cytotoxicity was measured using the dimethyl thiazolyl diphenyl (MTT) assay method. The medium was replaced with a fresh one with a 5 mg/mL MTT solution. After 4 h incubation time, the medium was removed, 150 µL dimethyl sulfoxide (DMSO) was added. The mixture was treated by ultrasound at 44 kHz for 3 min. The absorbance was measured on an EFOS 9305 microplate reader (Russia) at 492 nm with a reference wavelength of 620 nm. Cell viability was determined as the ratio of the sample’s absorbance to the control expressed as a percentage.

### 2.18. Statistical Analysis

Statistical data processing was performed by Statistica 6.0 software (StatSoft Inc., Tulsa, OK, USA). The normality of a distribution of results was shown using the Shapiro–Wilk test. The significance of differences between groups was assessed using Student’s *t*-test and one-way analysis of variance (ANOVA). The differences were considered statistically significant at *p* < 0.05.

## 3. Results and Discussion

### 3.1. Optimization of the Synthesis of ZnO NPs and Development of a Method for Designing Nanocomposites of BC and ZnO NPs

In this work, we used a sol-gel method for obtaining unprotected ZnO NPs exhibiting quantum-dots properties [41]. Protected ZnO NPs were obtained by synthesis from zinc acetate and lithium hydroxide in the presence of PEG 400 [42] and zinc nitrate and sodium hydroxide in the presence of trisamine.

Table 1 shows the sequence of preparation of various samples of nanocomposites of BC and ZnO NPs in film and powder form.

We studied BC-ZnO NPs composite films containing 1–30% of ZnO NPs. Powder X-ray diffractometry (PXRD) analysis was performed after drying samples stored in a desiccator.

The average particle size (diameter) was determined by PXRD according to the Scherrer Formula (2):

(2) $$D\;=\;\frac{k\times \lambda \;}{\beta \times cos\theta },$$

where *k* is the dimensionless particle shape factor, taken as 0.89; *λ* is the wavelength of X-ray radiation (0.154178 nm); *β* is the width of the reflex at half height in rad; and θ is the scattering angle in rad.

**Figure 1:**
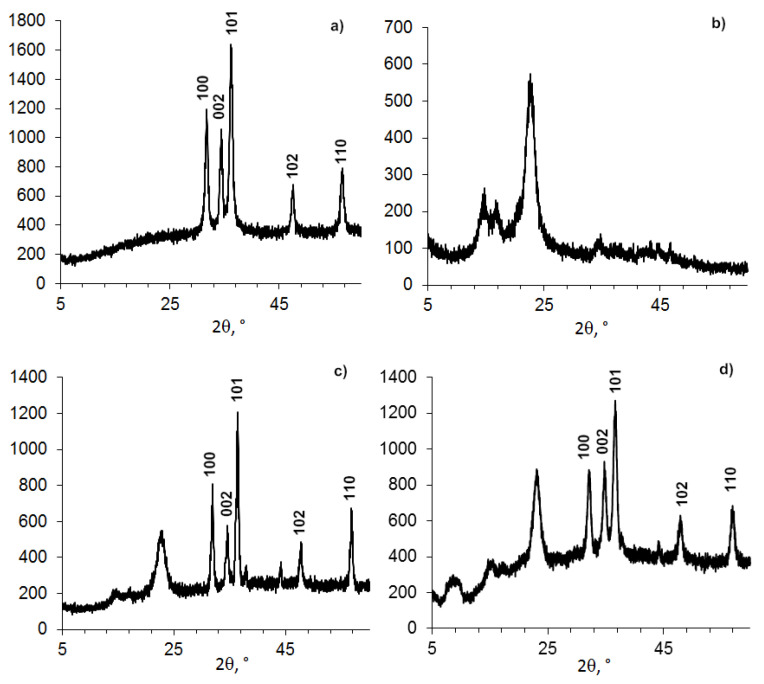
Powder XRD patterns of BC-ZnO samples: (**a**) ZnO NPs-PEG, (**b**) BC powder, (**c**) BC film–ZnO NPs, and (**d**) BC powder–ZnO NPs.

The data in Figure 1a and identification by the database show that the obtained protected ZnO NPs and ZnO NPs immobilized into the BC matrix had a hexagonal wurtzite shape with an average size of 11.5–18.71 nm (Table 2).

The effect of surfactants (cationic benzalkonium chloride or anionic sodium dodecyl sulfate) on the structure of the BC-ZnO NPs composites was insignificant. In all cases, a crystal hexagonal structure of wurtzite was formed in the BC matrix. A weak effect of cationic and anionic surfactants on the polymorphism of calcium carbonate (polymorph-calcite) during its precipitation on BC was noted in [53]. On the other hand, these authors obtained crystals of various shapes depending on the type and concentration of surfactants.

**Figure 2:**
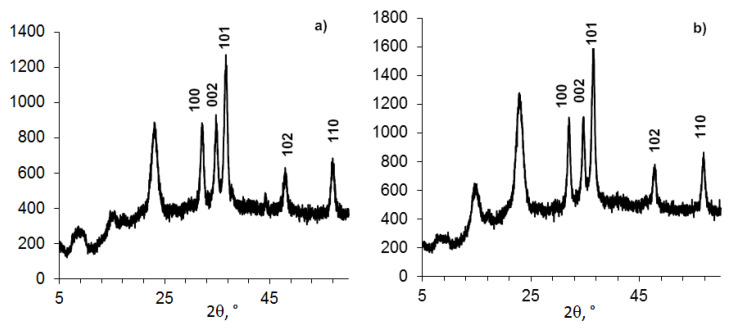
Powder XRD patterns of compositions (BC 0.5 g + ZnO NPs 0.15 g) samples, formed in different conditions in the presence of tris (**a**) or DDS-Na (**b**).

Changes in the nanocomposite were observed for the BC structure having an initial average size of 4–6 nm (Figure 1 and Figure 2, Table 2). The peak position with a maximum at 2θ = 22.96–23.08 degrees changed insignificantly, but the shape and position of the other two peaks inherent in crystalline BC had a different appearance. It can be assumed that the change in the structure of BC in the presence of ZnO NPs is due to the higher elasticity of BC (the ability of cellobiose units in the form of a “chair” to change their conformation) and smaller size compared to ZnO NPs.

The conditions (time, alkaline volume, reagent addition sequence) are indicated in the Table 3.

**Figure 3:**
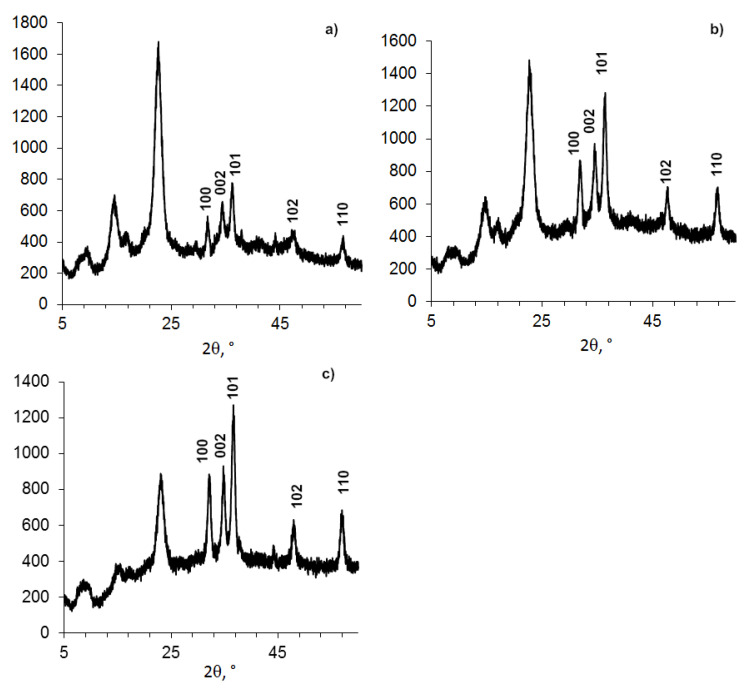
Powder XRD patterns of compositions (BC-ZnO NPs) samples, with concentrations of ZnO NPs: **a**). 6.6%; **b**). 16.9%; **c**). 26%.

This influence of ZnO NPs in the composition on the BC structure is indirectly confirmed by the study of composites BC-ZnO NPs, in which the concentration of ZnO NPs increases from 3.8% to 23.0% (Figure 3, Table 4). The BC peaks at a high concentration of ZnO NPs at small angles (2θ from 5° to 15°) appear indistinct.

The immobilization of betulin diphosphate (BDP) in BC can be made by swelling the cellulose in either an alcoholic BDP solution or an aqueous BDP sodium salt solution in the molar ratio trisamine: BDP = 4:1. After transferring the obtained BC-BDP films into a flat container, they were dried. To obtain complex combined composites BC-ZnO NPs-BDP, a suspension of an aqueous-alcoholic solution of ZnO NPs was sprayed onto the resulting BC-BDP film.

**Figure 4:**
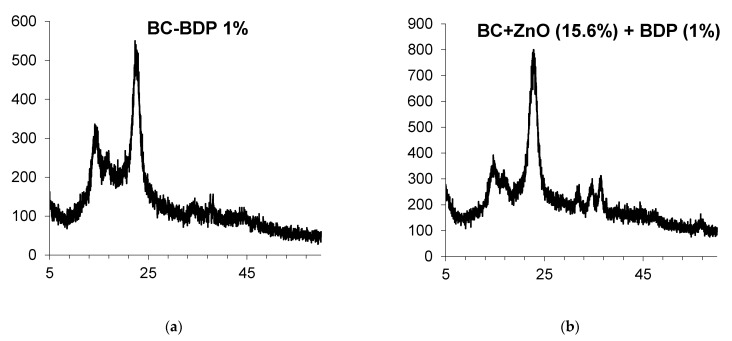
Powder XRD patterns of BC compositions: (**a**). BC-BDP (1%); (**b**). BC (DDS-Na 0.01%) + ZnO NPs+ BDP.

Powder X-ray diffractometry data are presented in Figure 4 and Table 5.

**Figure 5:**
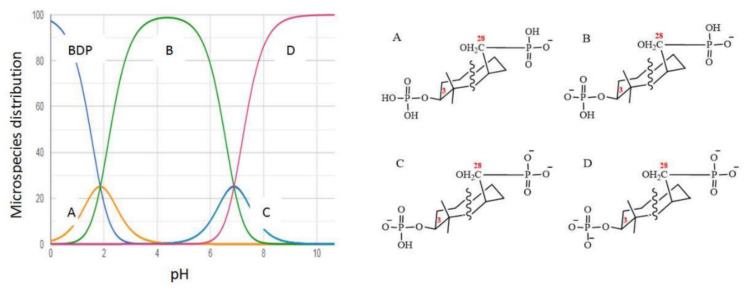
The dependence of BDP microspecies distributions (%) on pH calculated using Chemicalize program [54].

ZnO NPs were treated with BDP water:ethanol (1:1) previously for the decrease of possible aggregation. The zeta potential of ZnO NPs was equal to +15.9 mV in water:ethanol (1:1) solution but zeta potential of ZnO NPs modified by BDP was equal to −41.2 mV in water:ethanol (1:1) solution. The zeta potential changes can be explained by ionization of BDP phosphate groups in water:ethanol (1:1) solution (pH near 3.75) (Figure 5).

It was shown by electrospray ionization mass spectrometry at 180 °C in methanol that the molecular isotopic mass of BDP is equal to 602.314. It is seen that the spectrum contains one peak corresponding to an ion with a molecular weight of 601.3067 (the peak itself and the isotope distribution for this ion are shown in Figure S3). Consequently, BDP ionization occurs by eliminating only one proton in methanol, but two or more protons may be eliminated in an aqueous solution.

**Figure 6:**
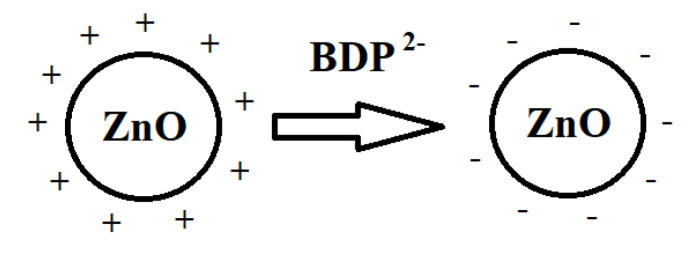
Schematic representation of the formation of negative charge.

Schematic representation of the BDP adsorption onto ZnO NPs’ surface from water:alcohol solution leads to the generation of negative charge of ZnO NPs’ surface as shown in Figure 6. Observed zeta potential value after BDP adsorption onto ZnO NPs surface suggests that aggregation is difficult.

Similar formation of negative charge onto ZnO NPs’ surface and an increase of dispersion stability in the presence of citric acid were described in the work [55].

If BC was previously wetted by BDP water:alcohol solution and then ZnO NPs are loaded into the BC-BDP system, the positive charge of ZnO NPs will be changed to negative, which will prevent agglomeration of colloid particles.

SSAs of ZnO NPs without BDP protection are equal to 35.2 ± 1.3 m^2^∙g^−1^. SSAs of ZnO NPs with BDP protection are equal to 40.5 ± 0.9 m^2^∙g^−1^.

**Figure 7:**
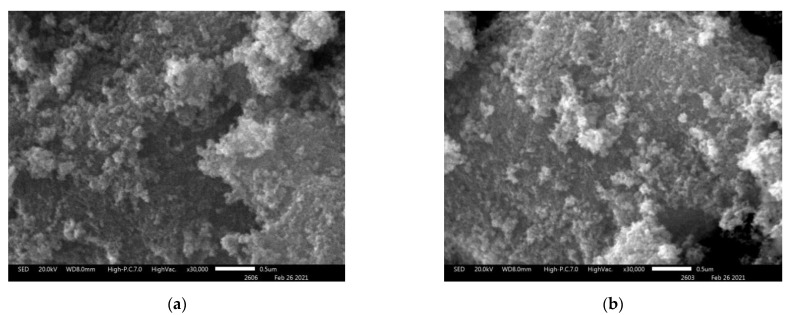
Scanning electron microscopy (SEM) images of ZnO NPs (**a**) and ZnO NPs modified by BDP (**b**), ×30,000.

Figure 7 shows that the SEM image of ZnO NPs modified by BDP (ZnO NPs-BDP) is similar to the SEM image of ZnO NPs. Nanoparticles sizes calculated by PXRD of ZnO NPs are close to ZnO NPs-BDP sizes (Table 2 and Table 5). This fact characterizes the absence of nanoparticles aggregation after modification.

The composition and structure of the composites based on BC were studied by IR spectral analysis, atomic absorption spectrophotometry, and complexometric titration of zinc oxide (Table 6, Figure S4).

**Figure 8:**
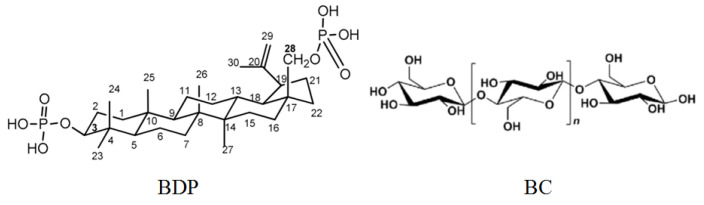
Formulas of betulin-3,28-diphosphate (BDP) and cellulose unit.

FTIR spectrum of BC-ZnO NPs composite has bands of ZnO NPs (ν 450 cm^−1^), intense C-O (hydroxyl) bands of BC (ν 1037–1200 cm^−1^) and intense CH, CH_2_ bands (ν 2900 cm^−1^). Moreover, the spectrum contains an intense band of stretching vibrations of OH groups in the region of 3355 cm^−1^. It can be noted that immobilization of BDP into the BC matrix does not change the shape of the PXRD patterns. In contrast, the inclusion of ZnO NPs in the composition significantly affected the spectra. Probably, BDP can adsorb on the surface of ZnO NPs due to the chemisorption binding of phosphate groups of BDP with Zn^2+^ ions. Besides, the “chair” conformational structures in the triterpenoid and cellulose unit promote a softer introduction of the triterpenoid into the BC structure (Figure 8).

The spectra of ZnO NPs contain an absorption band characteristic of ZnO NPs in the region of 450–500 cm^−1^ and hydrated Zn(H_2_O)_2_^2+^ ions in the region of 890–850 cm^−1^, as well as an absorption band corresponding to hydroxyl groups in the region of 3449–3377 cm^−1^. IR spectra of PEG-stabilized ZnO NPs have absorption bands characteristic of PEG (Figure S4).

The spectrum of BC contains bands of the monomeric unit of cellobiose (ν, cm^−1^): 596, 620, 642, 665, 744, 890, and 990 (7 CH-OH, 1 CH_2_-OH) [56]. The first five bands are moderately expressed, but the bands’ characteristic of stretching vibrations of alcohol C-O groups in the region of 1037–1200 cm^−1^ and stretching vibrations of CH and CH_2_ groups in the region of 3000–2900 cm^−1^ are intensely expressed. Furthermore, the band of stretching vibrations of OH-groups in the region of 3355 cm^−1^ is intensely expressed.

In the BC-ZnO NPs composite, the determining sign of zinc oxide nanoparticles’ appearance is the absorption band in the region of 450–470 cm^−1^. In the BC-ZnO NPs-BDP composite, in addition to the BC bands, a set of bands inherent in BDP appears (Figure S5).

The photoluminescence (PL) spectra of dispersions of ZnO NPs contained a weak blue emission absorption band λ_em_ = 372 nm, which characterizes the edge emission of quantum dots and the onset of ultraviolet absorption (Figure S2) [3]. In the UV spectra of either aqueous, alcohol, or both dispersions of ZnO NPs in the region of 340–370 nm, a band of abnormal light absorption (plasmon effect) appeared, which is characteristic of nanoparticles (Figure S2) [3].

**Figure 9:**
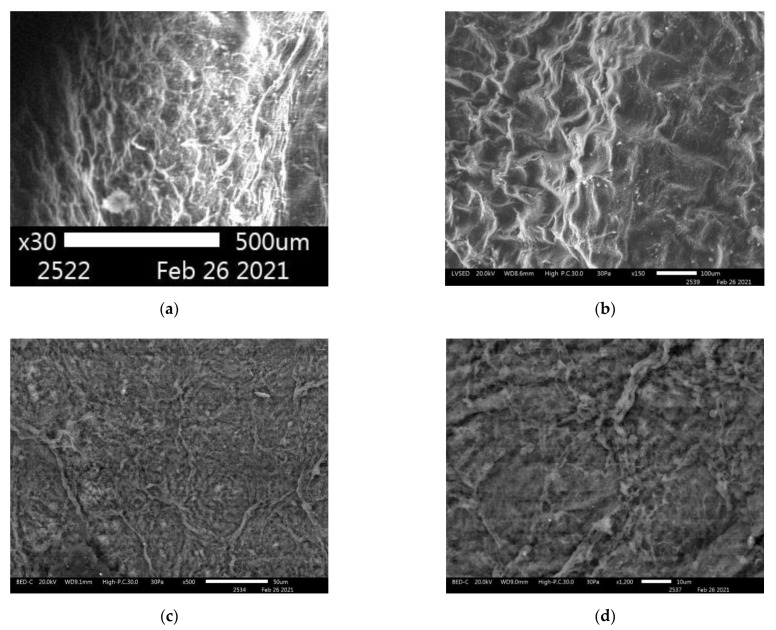
SEM images: BC hydrogel film ×30 (**a**) and ×150 (**b**); BC-ZnO NPs-BDP hydrogel film ×500 (**c**) and ×1200 (**d**).

Figure 9 shows that BC and BC-ZnO NPs-BDP hydrogels have different structures of nanocomposites after drying.

Thus, our results (PXRD, IR, UV, and PL spectroscopy) allow us to conclude that the composite material obtained based on bacterial cellulose contains zinc oxide nanoparticles.

Immobilization of BDP and ZnO NPs in BC without distorting the original structure of BDP, which activates collagen synthesis in the wound [3], is a prerequisite for using these composites as wound dressings.

### 3.2. Zinc Ions Release from BC-ZnO NPs-BDP Nanocomposite Films

**Figure 10:**
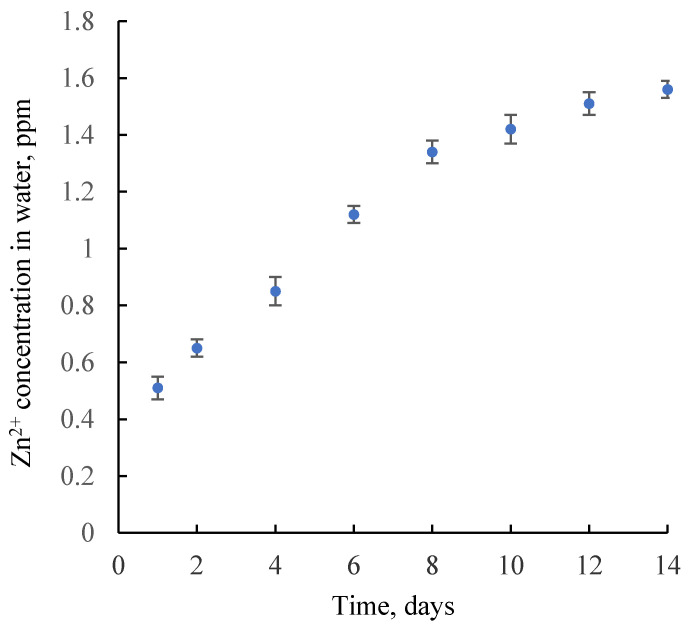
The assay of zinc ions released from BC-ZnO NPs-BDP nanocomposite films as membrane in water as a function of time using Franz vertical diffusion cells.

Assay of zinc ions released from BC-ZnO NPs-BDP nanocomposite films is important to understand the consequent toxicology of composites. The application of ZnO NPs containing composites in burn-wound healing might result in some health hazards of nanoparticles or free ions released in high quantity. The assay of zinc ions released from BC-ZnO NPs-BDP was estimated in water in Franz vertical diffusion cells (Figure S6) using the AAS or Inductively coupled plasma mass spectrometry (ICP) methods. Figure 10 shows a very small amount of zinc ions released during studied period. Release of zinc ions increased slowly with time and reached 1.56 ppm after day 14 of study. This fact is similar to results of work [15]. This slow release may be due to strong interaction of ZnO NPs with BDP being hydrophobic particles in a BC matrix.

### 3.3. Cell Viability under the Action of Wound Dressings and Their Biodegradability 

Cytotoxicity was estimated according to article [57] using the MTT test (Table 7). It has been shown that the influence of BDP, BC-BDP, and BC-ZnO NPs-BDP on cell viability was insignificant.

The biodegradability of BC may be varied depending on study conditions of degradation. Wang B. et al. [58] showed that BC biodegrades by cellulase in simulated body fluid in vitro and has compatibility in vivo. Their results demonstrate that the BC material had good in vitro and in vivo compatibility in the presence of cellulase. On the other hand, bacterial cellulose is stable in the absence of cellulase, and other factors induced hydrolysis of a linear chain of β(1→4) linked D-glucose units [59].

We showed that BC-ZnO NPs-BDP, BC-ZnO NPs, BC-BDP, and BC films placed into 0.9% NaCl solution for 21 days did not change pH value (pH 7.4 ± 0.2). We propose that pH value is stable during burn wound treatment by studied films when a 0.9% NaCl solution wets wound dressings.

### 3.4. Study of the Healing of a Thermal Burn Wound in an Experiment on Rats Treated with Agents Based on Bacterial Cellulose

BC-ZnO NPs-BDP films were closed on both sides with parchment and sealed in cellophane envelopes for biological studies.

A pharmaceutical composition with high wound healing activity containing ZnO NPs of a similar nature and size (wurtzite, 10–20 nm) was chosen as a reference drug [3]. The pharmaceutical composition, made in the oleogel form, consists of zinc oxide nanoparticles (5%), betulin (5%), betulin diphosphate (0.01%), and sunflower oil (up to 100%).

Four groups of animals were studied:

1. BC-ZnO NPs group: treatment with BC films with immobilized ZnO NPs (5%) (Figure S7).
2. BC-BDP group: treatment with BC films with immobilized BDP (0.01%) (Figure S8).
3. BC-ZnO NPs-BDP group: treatment with BC films with immobilized ZnO NPs (5%) and containing betulin diphosphate (1.5%) (Figure S9).
4. Control group: treatment with ZnO NPs-BDP oleogels (Figure S10).

Healing was studied using film composites based on bacterial cellulose (BC) with zinc oxide nanoparticles (ZnO NPs), BC and betulin diphosphate (BDP), and BC with ZnO NPs and BDP in the form of a film and gel, using 12 rats in each group. Postmortem studies were performed on days 3, 7, 10, and 21 using 3 rats each time.

Post-mortem examination after 3, 7, 10 and 21 days of treatment in all cases of using film composite materials revealed that the liver, kidneys, spleen, lungs, and heart are normal. The adrenal glands are slightly enlarged. On the 7th day, when the external dressings were changed, cellulose “ingrowth” into the wound was noted. The edges of the film outside the wound did not adhere to the skin surface.

The difference between the treatment with the BC-BDP composite film coating (Figure S8) is the uneven adhesion and, accordingly, the film’s ingrowth into the rat skin wound. In the presence of ZnO NPs, the film grows into the skin more uniformly.

The best skin state was shown in the experimental group treated by films BC-ZnO NPs containing 1% BDP. There was a tight adhesion of the film and ingrowth into the wound throughout the entire wound area (Figure S9).

Figure S10 shows photographs of rat wounds treated with oleogel.

Wound healing was monitored by wound area measurement. On the 3rd day of healing, the area of wounds increased insignificantly (by 2–5%), which is probably due to the development of burn disease in the animal. On day 21, the wound area treated with BC-ZnO NPs-BDP films was reduced by 34.3%, while when treated with ZnO NPs-BDP oleogel, a large decrease up to 40.6% was observed (Table 8).

**Figure 11:**
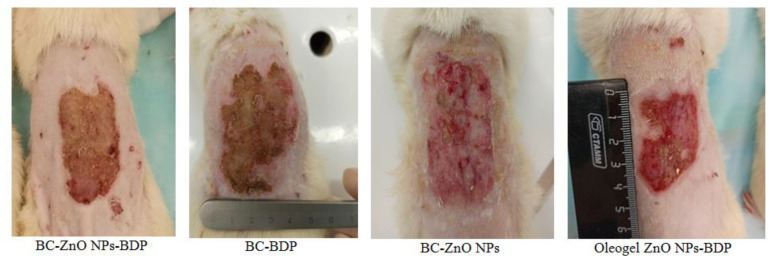
The burn wound state on day 21 after treatment.

Comparing the state of the burn wound on day 21 after treatment with the studied drugs showed that despite a smaller reduction in the wound area, the state of the burn wound when treated with BC-ZnO NPs-BDP films was the best (Figure 11). The healing process in rats is associated with skin tightening (construction), which is inhibited in the case of treatment with BC films, which partially grows into the wound. The skin in the wound area after treatment with oleogels looks inflamed, and small areas with suppuration were observed during treatment with BC-BDP films. Treatment with BC-ZnO NPs films in the absence of BDP reduced the wound area more slowly.

Morpho-histological studies have shown that on the third day after burn, all groups have a comparable histological picture. The histological structure of the epidermal tissue is disturbed. Destructive and degenerative processes in the upper layers of the dermis of animals were revealed. Infiltrative processes are accompanied by numerous foci of coagulation and total and subtotal necrosis. The end part, as well as the excretory duct of the sweat glands, are disturbed. The hair follicle is emptied and its own hair is missing. Coagulation necrosis encompasses both papillary and reticular dermis (Figure S11).

On the 7th day, common destructive changes in the epidermis and dermis persist in all groups. The epidermis is absent on almost the entire wound surface, except for the peripheral sections.

7 days after fixation of the BC-ZnO NPs, BC-BDP, and BC-ZnO NPs-BDP films, the scabs over the entire affected skin surface were almost completely desquamated. In the dermal plate’s deep layers and the hypodermis, the edema disappeared, and the blood vessels narrowed somewhat. The granulation tissue layer consisted of numerous newly formed thin collagen fibers. A large number of thin-walled small vessels of the capillary type and fibroblastic cells were revealed. A similar, however weaker, effect was observed in the group using the ZnO NPs-BDP-B oleogel (Figure S12).

On the 10th day of the experiment, animals of the ZnO NPs-BDP-B oleogel group in the affected zone revealed a wide band of granulation tissue with thin multidirectional collagen fibers. Granulation tissue with macrophages, lymphocytes, fibroblastic cells of varying degrees of maturity, and well-differentiated and poorly differentiated cells was observed. On the 10th day of the experiment, in animals of the BC-ZnO NPs-BDP group, the bottom of the burn wound was filled with granulations, signs of the transition of granulation tissue into connective tissue appeared, and vascular invasion was noted.

The skin’s epithelial layer was not restored in the BC-ZnO NPs and BC-BDP groups, even on the 10th day of the thermal burn zone experiment. A small zone of growing epithelium was determined from the periphery only. The exudate accumulation was revealed with its spread to the adjacent granulation tissue, which was represented by thin connective tissue fibers (Figure S13).

By the 21st day of the experiment, in animals of the BC-ZnO NPs, BC-BDP groups, the formed dermal plate was mostly represented by mature fibrous connective tissue, consisting of collagen bundle fibers infiltrated by fibroblastic cells. An even layer of the stratified keratinizing epithelium covering it lay on a clearly expressed thin basement membrane.

By the 21st day of the experiment, the BC-ZnO NPs-BDP group showed signs of parallel processes of maturation of granulation tissue and epithelialization of the regeneration. In the ZnO NPs-BDP-B oleogel group, partial peripheral epithelialization can be traced (Figure S14).

Based on the morphophysiological studies of tissue during treatment with various drugs based on composite materials (BC-ZnO NPs, BC-BDP, BC-ZnO NPs-BDP) and oleogel (ZnO NPs-BDP-B), additionally protected by dressings, we can draw the following conclusions:

1. At the initial stage, the healing of a thermal burn with the use of composite materials based on BC: BC-ZnO NPs, BC-BDP, BC-ZnO NPs-BDP, and ZnO NPs-BDP-B oleogel is similar. This process is characterized by destructive changes in the skin with signs of complications of the reparative process, which is manifested by uneven maturation of granulation tissue with the presence of foci of inflammation and secondary stromal necrosis and signs of impaired proliferation, differentiation, and keratinization of the epidermis in the form of focal hyperplasia of the epidermis with symptoms of acanthosis and hyperkeratosis.
2. The use of ZnO NPs-BDP-B oleogel, as a rule, limits the destructive changes caused by thermal damage within the epidermis and dermis. The use of ZnO NPs-BDP-B oleogel promotes granulation tissue formation, its maturation, and epithelialization. Simultaneously, the reparative process during treatment with oleogel proceeds to a lesser extent than the BC-ZnO NPs, BC-BDP and BC-ZnO NPs-BDP animal groups, complicated by the secondary stromal necrosis and inflammation foci of the regenerate and adjacent tissues.
3. Reparative processes when using BC composite materials proceed more intensively than using oleogel due to the destructive process’s limitation within the epidermis and dermis. Besides, the purulent-necrotic complications are absent in the process of defect healing by BC composites. In this case, the reparative process ensures the earlier appearance and uniform maturation of young connective tissue with the normalization of proliferation and differentiation of epidermal cells.

### 3.5. Investigation of Microhemocirculation in the Skin of Rats after a Burn and during Treatment with Agents Containing Bacterial Cellulose

Microcirculation intensity also assessed the burn wound’s state by using laser Doppler flowmetry (LDF), where laser radiation is reflected from the tissue. This method is based on the Doppler frequency shift depending on erythrocytes’ speed in arterioles, capillaries, and venules [24].

There are various types of oscillations with different amplitudes, characterizing changes in microhemocirculation, to which the endothelial (E), myogenic (M), and neurogenic (N) frequency ranges, which determine the mechanisms of regulation of the lumen of blood vessels and their tone, contribute.

The information obtained by the LDF method makes it possible to measure the rate of movement of erythrocytes and the amount of tissue oxygenation in the blood flow (perfusion). Small fluctuations in oxygenation of a burn wound’s tissue generally reflected that metabolic changes can also be evaluated.

The average amplitude of the reflected signal in the microhemocirculatory flow makes it possible to obtain the average Doppler frequency shift over all the probed areas. We analyzed the amplitude-frequency spectrum using the wavelet transform [60].

The result of flowmetry can be represented by an indicator of microcirculation (PM), proportional to the number and average speed of erythrocytes in the probed volume and is expressed in arbitrary units (perfusion units).

Figure S15 shows typical LDF-grams and their wavelet spectra obtained immediately after the burning (day 0) and on day 10 after burning. Immediately after the burn, microcirculation and respiration (oxygenation) are disturbed in all areas of the microhemocirculatory zone (Figure S15a). On the 10th day after the burn, oxygenation of the microvasculature tissue (respiratory and cardiac bands of wavelet spectra) normalizes and reaches the maximum value. At the same time, all other LDF signals decrease (Figure S15b).

The dynamics of perfusion change as different microhemocirculatory indexes calculated by neurogenic, myogenic, cardiac, endothelial, and respiratory bands in wavelet spectra are shown in Table 9 and Table 10.

Thus, the spectroscopic data of the LDF method after wavelet transformation characterize the recovery of the velocity of erythrocytes and the value of tissue oxygen saturation in the blood flow (perfusion) after treatment with the proposed BC-ZnO NPs-BDP wound dressings.

### 3.6. Antioxidant Properties of BC-ZnO NPs-BDP Nanocomposites

**Figure 12:**
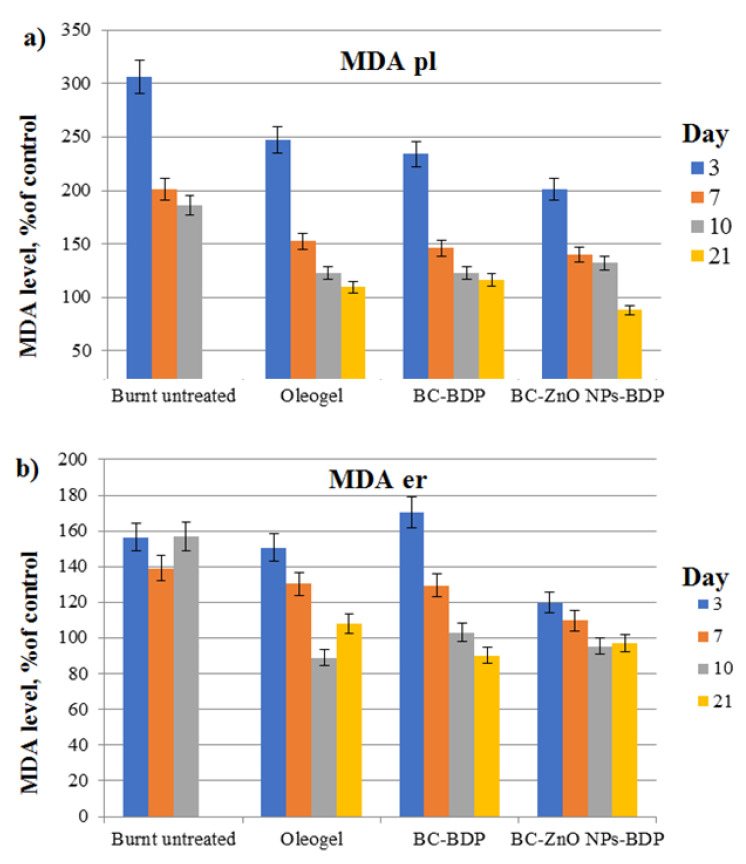
Diagram of changes in MDA concentration in plasma and blood erythrocytes (μmol/L) during treatment with the studied drugs (% of control). Number of experiment replications was equal to 3. *p* < 0.001. MDA pl (**a**) and MDA er (**b**) mean the level of MDA in blood plasma and erythrocytes.

**Figure 13:**
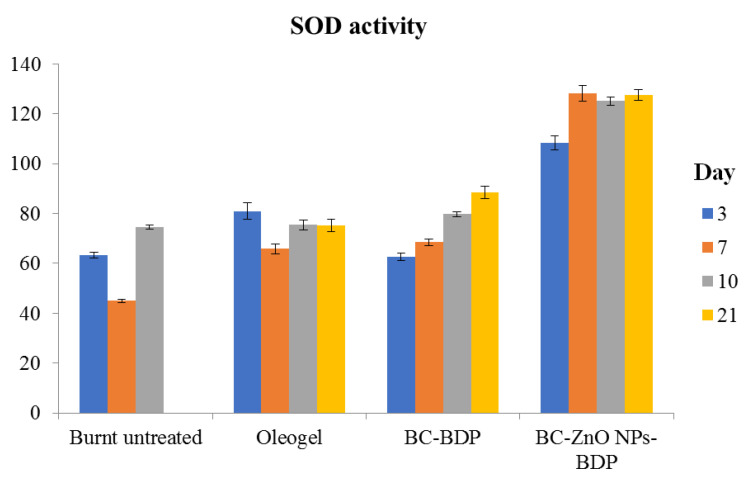
Diagram of changes in superoxide dismutase (SOD) activity during treatment with the studied agents (% of control), 100%—1037.762 ± 21.351% ng min^−1^ mg protein^−1^. The number of experiment replications was equal to 3. *p* < 0.001.

Main oxidative stress markers are the level of malondialdehyde (MDA) in blood plasma and erythrocytes and the level of activity of antioxidant defense enzymes such as superoxide dismutase and catalase (Figure 12 and Figure 13, Table 11).

From the study results, it follows that the treatment of a burn wound with the studied drugs normalizes the MDA level by 7, 10, and 21 days.

The data in Figure 13 (SOD) and Table 11 (catalase) follows that the studied drugs, in particular wound dressings, exhibit antioxidant properties. On the 21st day, the SOD activity increases by 27% compared to the intact control, and the catalase activity practically reaches the intact control.

The significant effect of zinc oxide nanoparticles on the activity of SOD can be partially explained by the activation of the zinc in the active site of CuZn-SOD, which has extremely high stability and high activity of superoxide dismutation.

The zinc role, which does not change the oxidation state in redox protection against oxidative stress, is more complex than copper, which interacts according to the Fenton reaction. In this context, zinc can be considered an “indirect” antioxidant-a modifier of biological redox reactions. The antioxidant effect of zinc is mainly due to its ability to induce a stress response in terms of (1) stimulation of MTF-1-dependent transcription and (2) activation of stress-sensitive signalling cascades MAPK and PI3K/Akt. Besides, zinc’s antioxidant effect is associated with the stabilization of protein thiols (enzymes, zinc fingers, metallothioneins) [16].

Potential mechanisms of action of zinc can be explained by antagonism of redox transition metals, such as iron or copper, and prevention of oxidation of sulfhydryl groups in proteins. Thiol groups are stabilized by zinc, which protects the enzyme or other protein from inactivation caused by oxidative stress. The action of zinc as an antioxidant in the case of metallothioneins is to regulate their metabolism. In turn, zinc deficiency leads to a decrease in the protection of sulfhydryl groups and an increase in reactive oxygen species (ROS). Excessive levels of zinc can act as prooxidants, causing a decrease in the level of CuZn-SOD and other important metalloenzymes in erythrocytes. In general, biochemical studies of many diseases, such as diabetes mellitus, have confirmed that optimal zinc levels are a prerequisite for maintaining normal oxidative metabolism [16].

Thus, our studies have shown that by day 21, wound dressings based on BC and ZnO NPs with immobilized BDP lead to the restoration of the antioxidant balance in the blood of rats with thermal injury both by reducing the intensity of free radical oxidation and by increasing the antioxidant enzyme protection.

## 4. Conclusions

A nanosized BC hydrogel film has shown itself as a promising matrix for the sorption of anti-burn BDP and ZnO NPs modified with BDP. Zinc oxide nanoparticles protected by BDP do not aggregate in both the individual state and in the nanocomposite. Moreover, zinc ions release and cell survival results are the arguments for the decrease in the protein corona formation on ZnO NPs surfaces. The results of biological studies carried out by us on rats (wound area, improved healing, biochemical parameters and microcirculation, morphological picture) indirectly confirm the perspective of medical use of ZnO NPs modified with betulin diphosphate as a component of nanocomposites. In general, it creates the prerequisites for high stability and uniformity of the final BC-Zn NPs-BDP nanomaterial. Our cell viability data show that the BC-Zn NPs-BDP nanocomposite toxicity is reduced due to the protection of zinc oxide nanoparticles by the BDP modification. It was found that BC-Zn NPs-BDP demonstrated a set of properties necessary for wound dressings to treat burns in in vivo experiments. These coatings can accelerate wound healing due to the inclusion of several mechanisms: regulation of oxygenation and microcirculation, reduction of hypoxia in a burn wound, reduction of oxidative stress resulting from the development of burn disease, and activation of antioxidant enzymatic protection.

## Figures and Tables

**Table 1:** Preparation procedure of BC (bacterial cellulose)-ZnO (nanoparticles) NPs composites.

BC Type	1 Step	2 Step	3 Step
film	soaking, 3 h in 1% trisamine solution, then 5 h in aq. solution of Zn(NO_3_)_2_∙4 H_2_O	BC film removing and transfer into shallow container with 0.1 M solution of NaOH for 10 min at 50 °C	rinse with distilled water until the composite pH became neutral, then drying
film	soaking, 3 h in 1% trisamine solution or DDS-Na or benzalkonium chloride for 30 min	BC film removing and transfer into shallow container, then spraying of ZnO NPs water-alcohol dispersion onto film surface	drying
powder	soaking, 3 h in 1% trisamine solution or DDS-Na or benzalkonium chloride for 30 min, sonification	transfer into vessel, then adding of ZnO NPs water-alcohol dispersion, then sonification	separation solid phase, rinse with distilled water until the composite pH became neutral, then drying

**Table 2:** Data of powder XRD patterns of BC-ZnO NPs samples (Figure 1).

Figure Number	Sample	Composition (Treatment ^1^)	2θ, Degree	D, nm	Structure Signal
a	1	ZnO NPs-PEG	36.18	14.040	ZnO (wurtzite)
			47.60	11.562	
			56.54	12.285	
b	2	BC	22.96	4.749	BC
c	3	BC (film)–ZnO NPs	22.68	4.601	BC
			36.38	18.719	ZnO (wurtzite)
			47.80	18.284	
			56.84	15.725	
d	4	BC–ZnO NPs	23.08	5.520	BC
			36.64	10.620	ZnO (wurtzite)
-	-	ZnO NPs ^2^	31.76	11.600	ZnO (wurtzite)
			34.36	17.500	
			36.22	11.700	

^1^ Ultrasound treatment. ^2^ Data of our samples protected by betulin diphosphate (BDP) were published in [3].

**Table 3:** The dependence of the structure of BC-ZnO NPs composition (23% of ZnO) on formation conditions (Figure 2).

Figure Number	Sample	Procedure	ZnO NPs Content, g in 0.5 g BC (%), *n* = 3		2θ, Degree	D, nm	Signal
			Added	Found (AAS)			
a	5	1. Preparation of ZnO NPs dispersion in 1% tris aqueous solution (25 mL) at pH 10. 2. Ultrasound treatment 3. BC addition to dispersion under sonification 4. Storage for 1 h 5. Filtration and drying at 120 °C	0.150 (23.0 ± 0.1%)	(26.0 ± 0.2)%	22.96	5.88	BC
					36.64	11.19	ZnO
b	6	1. Preparation of BC water-ethanol dispersion in the presence of 0.01% DDS-Na aqueous solution (0.1 mL) at pH 10. 2. Ultrasound treatment 3. ZnO NPs addition to dispersion under sonification 4. Storage for 1 h 5. Filtration and drying at 120 °C	0.150 (23.0 ± 0.1%)	(22.6 ± 0.2)%	22.74	5.10	BC
					36.44	12.28	ZnO

**Table 4:** Data of Figure 3.

Figure Number	Sample	ZnO NPs Content, g in 0.5 g BC (%), *n* = 3		2θ, Degree	D, nm	Signal
		Added	Found (AAS)			
a	7	0.025 (3.8%)	(6.6 ± 0.2)%	22.64	6.33	BC
				36.32	13.49	ZnO
b	8	0.075 (11.5%)	(16.9 ± 0.2)%	22.68	5.37	BC
				36.42	10.62	ZnO
c	5	0.150 (23.0%)	(26 ± 0.2)%	22.96	5.88	BC
				36.64	11.19	ZnO

**Table 5:** Data of Powder XRD patterns of BC-ZnO NPs samples (Figure 4).

Figure Number	Sample	Composition (Treatment ^1^)	2θ, Degree	D, nm	Signal
a	9	BC-BDP (1%)	22.48	6.25	BC
b	10	BC (DDS-Na 0.01%) + ZnO NPs + BDP	22.64	4.40	BC
			36.20	12.16	ZnO

^1^ Ultrasound treatment.

**Table 6:** Composition and FTIR spectral characteristics of ZnO NPs and bacterial cellulose samples (Figure S4).

Composition	ZnO, %		ν, sm^−1^ 420–500
	Ad.	Found, *n* = 3	
ZnO NPs	100	100	450–467 (υ ZnO NPs)
BC	-	-	596, 620, 642, 665, 899, 1037, 1062, 1200, 2366, 2900 (CH, CH_2_), 3347, 3355 (OH)
BC-ZnO NPs	16.67	17.8 ± 0.2	450–470
BC +0.01% DDS-Na + ZnO NPs	16.67	16.2 ± 0.3	473 (ZnO NPs),

**Table 7:** Cell viability under the action of dispersions of studied wound dressings (BC-BDP, BC-ZnO NPs-BDP) and BDP (*n* = 3, *p* < 0.01).

Concentration of Studied Dispersion, μg∙mL^−1^	Cell Viability, % of Control (Cells Only)			
	BC	BDP	BC-BDP	BC-ZnO NPs-BDP
0	101.0 ± 3.9	100.9 ± 2.0	100.4 ± 5.1	99.3 ± 4.5
12.5	102.2 ± 3.1	94.0 ± 3.1	105.2 ± 3.3	101.2 ± 3.3
25.0	105.8 ± 1.7	92.9 ± 1.8	102.6 ± 2.8	109.4 ± 5.2
50.0	104.0 ± 7.3	105.3 ± 8.4	93.8 ± 2.1	103.5 ± 1.0
100.0	110.3 ± 3.6	109.2 ± 2.1	107.7 ± 6.3	105.4 ± 5.9

**Table 8:** Wound area changes during treatment with BC, BC-ZnO NPs-BDP (film), and ZnO NPs-BDP oleogel (*n* = 5, *p* < 0.001).

Group	Wound Area, cm^2^				
	0 Day	3 Day	7 Day	10 Day	21 Day
Burn untreated	21.643 ± 0.920	21.409 ± 0.835	20.772 ± 0.659	20.438 ± 0.899	17.492 ± 1.015
BC	21.319 ± 0.932	21.282 ± 1.085	20.639 ± 0.599	19.921 ± 1.331	17.695 ± 2.532
BC-ZnO NPs-BDP (film)	21.846 ± 0.679	21.645 ± 1.294	19.953 ± 0.291	19.455 ± 0.544	14.343 ± 0.756
Oleogel ZnO NPs-BDP	21.599 ± 0.628	20.366 ± 0.306	18.798 ± 0.307	19.897 ± 0.313	12.825 ± 0.311

**Table 9:** Results of Doppler flowmetry of blood microcirculation in a burn wound under the action of bacterial cellulose wound dressings (*p* < 0.001).

Time, Day	Group	MI, perf. un. ^1^	E, arb. un. ^2^	N, arb. un.	M, arb. un.	R, arb. un.	C, arb. un.
N/A	healthy	13.26 ± 1.21	6.63 ± 0.60	6.14 ± 0.56	7.64 ± 0.69	22.72 ± 2.07	11.80 ± 1.07
0	burnt	6.31 ± 0.57	12.63 ± 1.15	14.68 ± 1.33	15.66 ± 1.42	14.14 ± 1.29	9.66 ± 0.88
3		12.96 ± 1.18	5.25 ± 0.48	6.73 ± 0.61	6.92 ± 0.63	24.95 ± 2.27	12.05 ± 1.10
7		15.16 ± 1.38	6.05 ± 0.55	6.76 ± 0.61	8.61 ± 0.78	18.99 ± 1.73	11.53 ± 1.05
10		14.86 ± 1.35	6.67 ± 0.61	6.43 ± 0.58	7.94 ± 0.72	21.07 ± 1.92	10.72 ± 0.97
21		12.17 ± 1.11	7.34 ± 0.67	7.21 ± 0.66	6.13 ± 0.56	20.13 ± 1.83	9.43 ± 0.86

^1^ perf. un.: perfusion units, ^2^ arb. un.: arbitrage units, E: endothelial, N: neurogenic, M: myogenic, R: respiratory and C: cardiac oscillations.

**Table 10:** Results of Doppler flowmetry of blood microcirculation in a burn wound under the action of control oleogel (*p* < 0.001).

Time, Day	Group	MI, perf. un. ^1^	E, arb. un. ^2^	N, arb. un.	M, arb. un.	R, arb. un.	C, arb. un.
N/A	healthy	13.26 ± 1.21	6.63 ± 0.60	6.14 ± 0.56	7.64 ± 0.69	22.72 ± 2.07	11.80 ± 1.07
0	burnt	6.31 ± 0.57	12.63 ± 1.15	14.68 ± 1.33	15.66 ± 1.42	14.14 ± 1.29	9.66 ± 0.88
3		11.47 ± 1.04	5.69 ± 0.52	6.51 ± 0.59	7.28 ± 0.66	27.87 ± 2.53	12.53 ± 1.14
7		13.05 ± 1.19	6.00 ± 0.55	6.82 ± 0.62	7.81 ± 0.71	23.71 ± 2.16	10.42 ± 0.95
10		13.19 ± 1.20	5.66 ± 0.51	7.17 ± 0.65	7.56 ± 0.69	23.08 ± 2.10	9.40 ± 0.85
21		13.20 ± 1.20	5.76 ± 0.52	7.78 ± 0.71	6.85 ± 0.62	21.23 ± 1.93	10.47 ± 0.95

^1^ perf. un.: perfusion units, ^2^ arb. un.: arbitrage units, E: endothelial, N: neurogenic, M: myogenic, R: respiratory and C: cardiac oscillations.

**Table 11:** Catalase activity under the treatment with the studied agents (% of control) ^1,2^.

τ, Day	Catalase Activity, % of Control			
	Burnt Untreated	Oleogel	BC-BDP	BC-ZnO NPs-BDP
3	47.98 ± 1.16	50.03 ± 1.85	57.50 ± 0.82	53.11 ± 1.81
7	60.13 ± 1.78	50.71 ± 1.09	50.77 ± 0.66	86.71 ± 3.84
10	51.89 ± 2.54	59.38 ± 2.08	58.90 ± 1.11	60.41 ± 0.98
21	N/A	62.39 ± 1.62	65.84 ± 0.42	92.49 ± 3.02

^1^ 100%—39.512 ± 0.903 μmol H_2_O_2_ min^−1^ mg protein^−1^, ^2^ The number of experiment replications was equal to 3. *p* < 0.001.

## Data Availability

Not applicable.

## Supplementary Materials

The following are available online at https://www.mdpi.com/2079-4991/11/3/713/s1, Figure S1: ^13^C NMR spectrum of solid bacterial cellulose, Figure S2: Fluorescence spectra of 27.2 mg% dispersions of ZnO NPs (a); UV spectrum of ZnO NPs in ethanol, 27.2 mg/%, blanc–ethanol (b), Figure S3: MS spectrum of BDP, Figure S4: FTIR spectra characteristics of ZnO NPs, bacterial cellulose (aerosol) samples and aerosol bacterial cellulose with immobilized betulin diphosphate under various conditions, Figure S5: FTIR spectra characteristics of BDP, BC + BDP (1%) and BC + BDP (1%) +ZnO (5%), Figure S6: Scheme of the Franz vertical diffusion cell, Figure S7: Treatment by BC-ZnO NPs composite, Figure S8: Treatment by BC-BDP composite, Figure S9: Treatment by BC-ZnO NPs-DBP composite, Figure S10: Treatment by ZnO NPs-BDP-B oleogel, Figure S11: Tissue state by morphological and histological examination (3 Day, hematoxylin-eosin, ×600), Figure S12: Tissue state by morphological and histological examination (7 Day, hematoxylin-eosin, ×600), Figure S13: Tissue state by morphological and histological examination (10 Day, hematoxylin-eosin, ×600), Figure S14: Tissue state by morphological and histological examination (21 Day, hematoxylin-eosin, ×600), Figure S15: Typical LDF grams and the corresponding wavelet spectra obtained immediately after the burning (0 day) and on day 10 after burning on the wounds of rats. E: endothelial, N: neurogenic; M: myogenic, R: respiratory, and C: cardiac oscillations.
