# Supplementary material for: Wound Healing Composite Materials of Bacterial Cellulose and Zinc Oxide Nanoparticles with Immobilized Betulin Diphosphate
Source: Nanomaterials (Basel). 2021 Mar 12;11(3):713. doi: 10.3390/nano11030713 (PMC8000300; doi:10.3390/nano11030713)
Supplement: Supplementary file 1 [file nanomaterials-11-00713-s001.pdf]

nanomaterials

Article

Wound Healing Composite Materials of Bacterial Cellulose and Zinc Oxide Nanoparticles with Immobilized Betulin Diphosphate

Nina Melnikova [1,*], Alexander Knyazev [1], Viktor Nikolskiy [2], Peter Peretyagin [3], Kseniya Belyaeva [3], Natalia Nazarova [4], Elena Liyaskina [4], Darina Malygina [5] and Viktor Revin [4]

[1] Faculty of Chemistry, Lobachevsky University, 23/5 Gagarin Av., 603950 Nizhny Novgorod, Russia; knyazevav@gmail.com
[2] Nizhni Novgorod Regional Clinical Hospital named after N.A. Semashko, 190 Rodionova str., 603126 Nizhny Novgorod, Russia; viktor22031@yandex.ru
[3] Department of Experimental Medicine, Privolzhsky Research Medical University, 10/1 Minin sq., 603950 Nizhny Novgorod, Russia; peretyaginpv@gmail.com (P.P.); skoln94@mail.ru (K.B.)
[4] Department of Biotechnology, Bioengineering and Biochemistry, National Research Ogarev Mordovia State University, 68 Bolshevistskaya str., 430005 Saransk, Russia; fac-bio@adm.mrsu.ru (N.N.); liyaskina@yandex.ru (E.L.); revinvv2010@yandex.ru (V.R.)
[5] Department of Pharmaceutical Chemistry, Privolzhsky Research Medical University, 10/1 Minin sq., 603950 Nizhny Novgorod, Russia; mds73@yandex.ru
* Correspondence: melnikovanb@gmail.com; Tel.: +79023092298

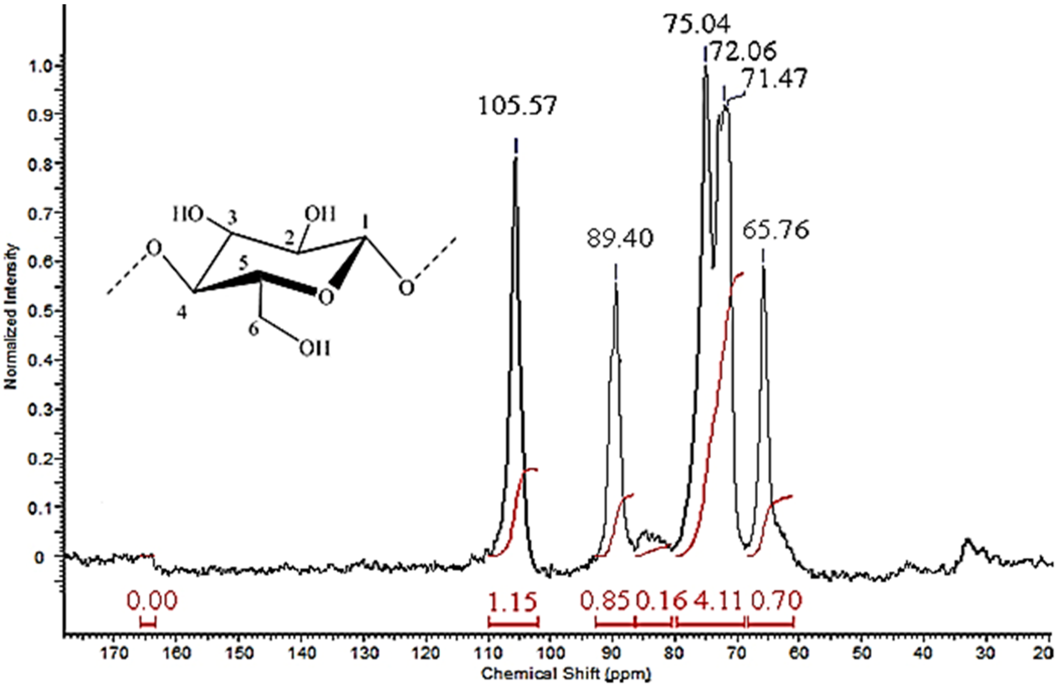

Figure S1. ^{13}C NMR spectrum of solid bacterial cellulose.

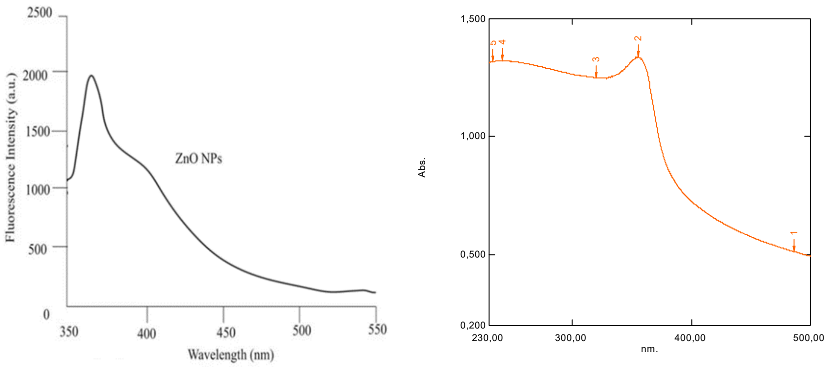

(**a**) (**b**)

Figure S2. Fluorescence spectra of 27.2 mg% dispersions of ZnO NPs (**a**); UV spectrum of ZnO NPs in ethanol, 27.2 mg/%, blanc – ethanol (**b**).

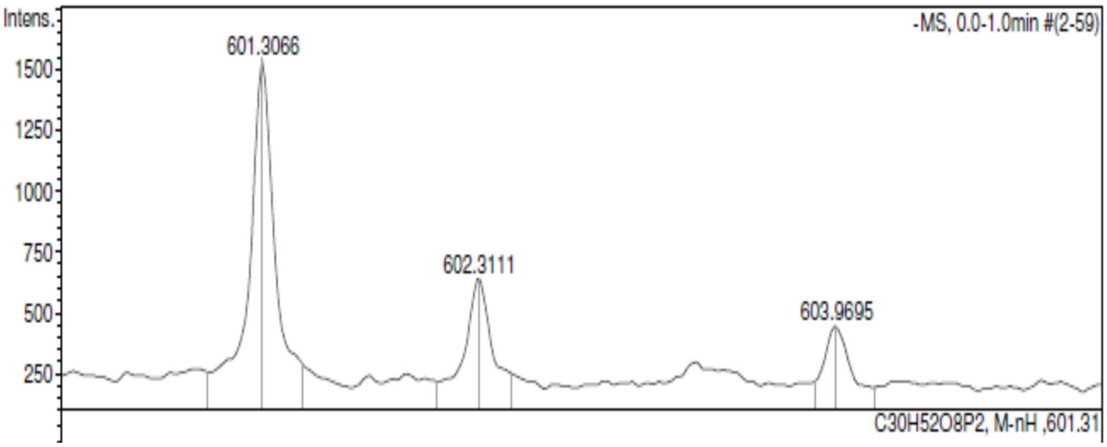

Figure S3. MS spectrum of BDP.

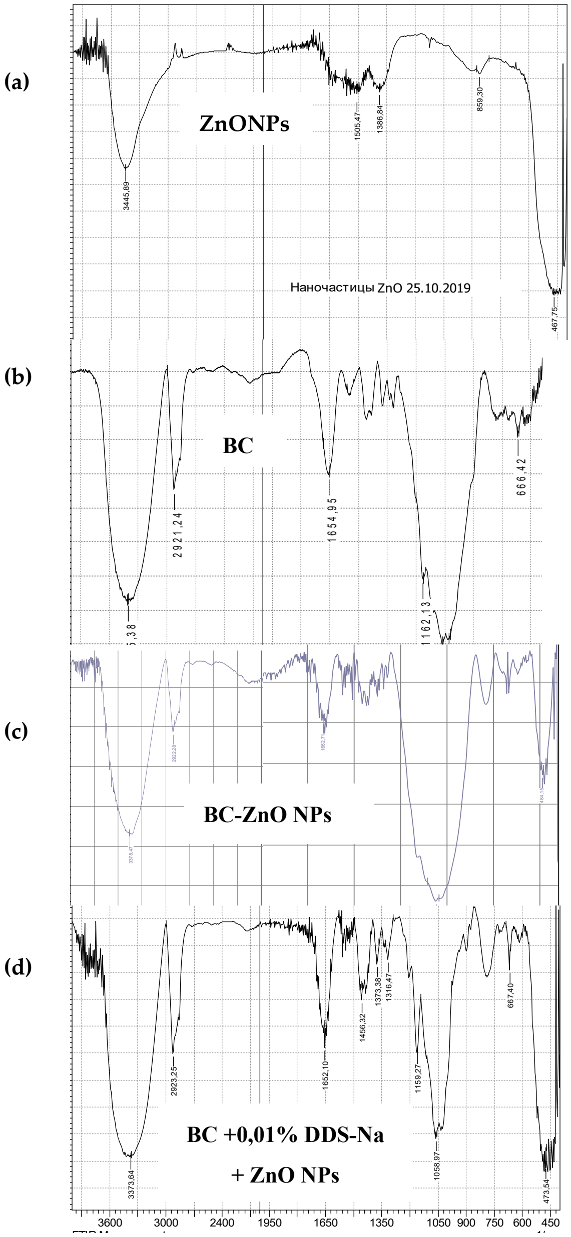

Figure S4. FTIR spectra characteristics of ZnO NPs (a), bacterial cellulose (b) samples and aerosol bacterial cellulose with immobilized ZnO NPs (c), BC + 0,01% DDS-Na + ZnO NPs (d).

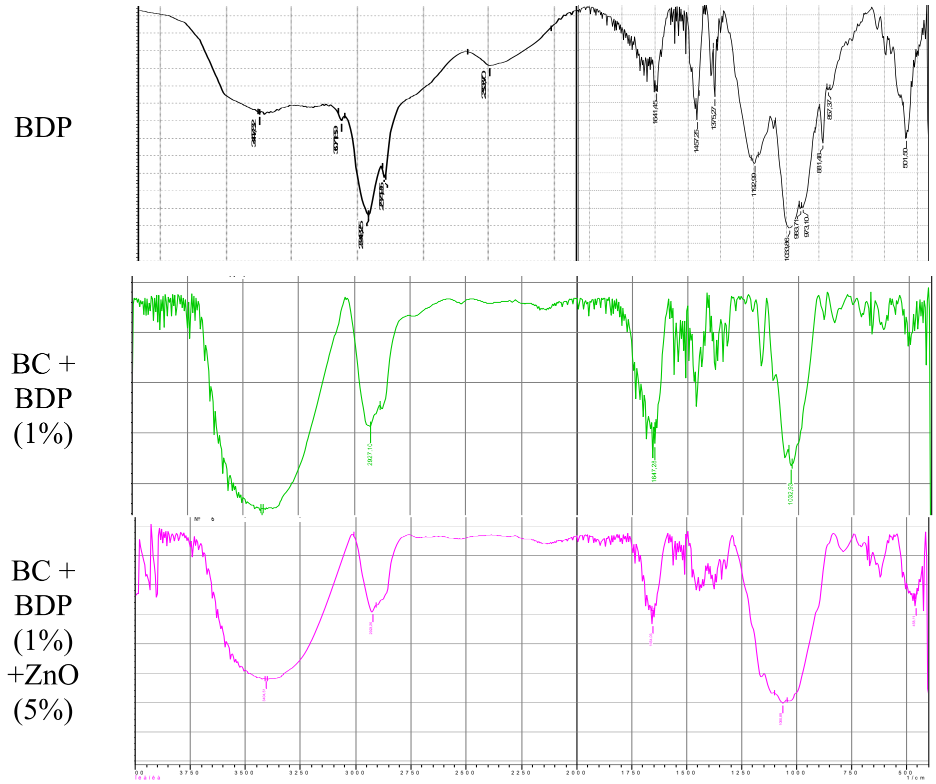

Figure S5. FTIR spectra characteristics of BDP, BC + BDP (1%) and BC + BDP (1%) +ZnO (5%).

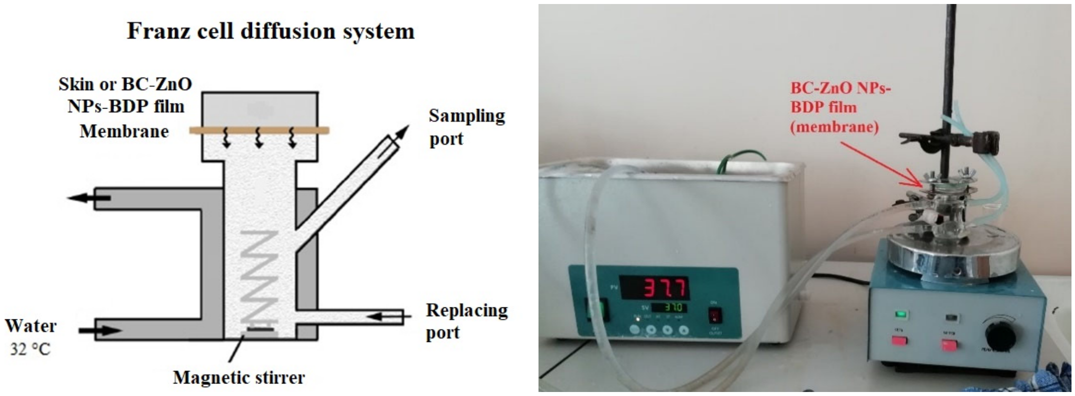

Figure S6. Scheme of the Franz vertical diffusion cell.

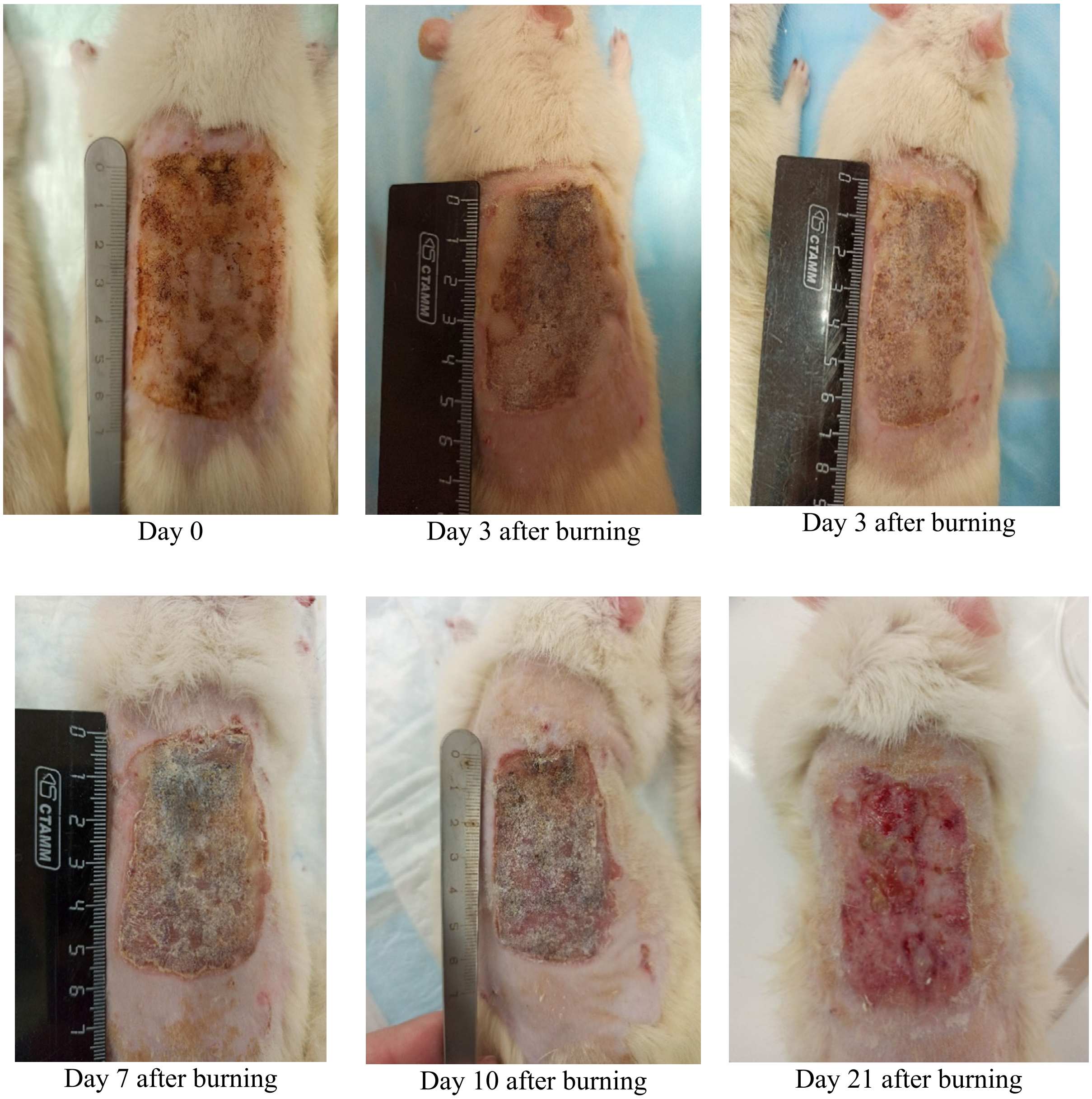

Figure S7. Treatment by BC-ZnO NPs composite.

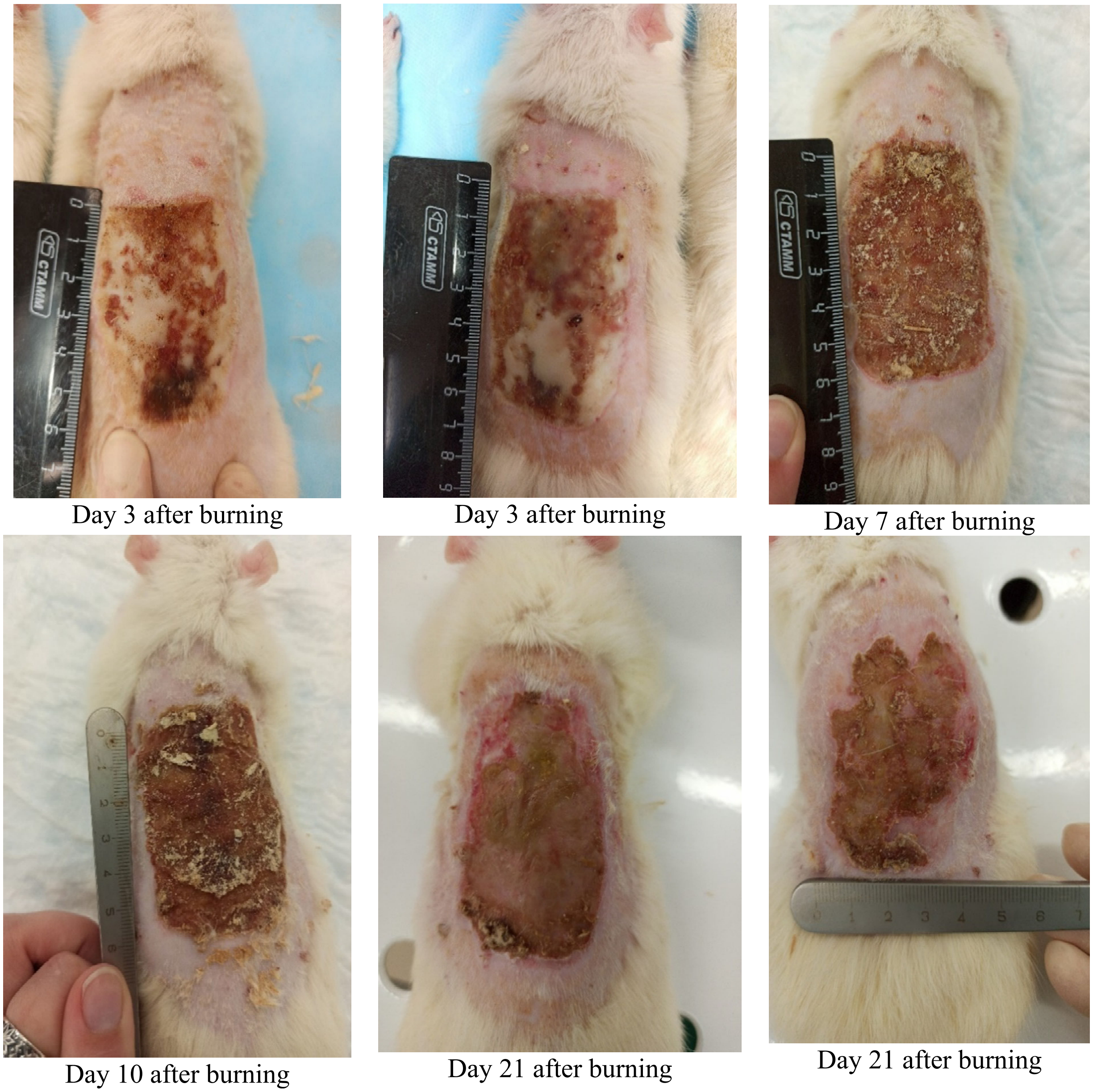

Figure S8. Treatment by BC-BDP composite.

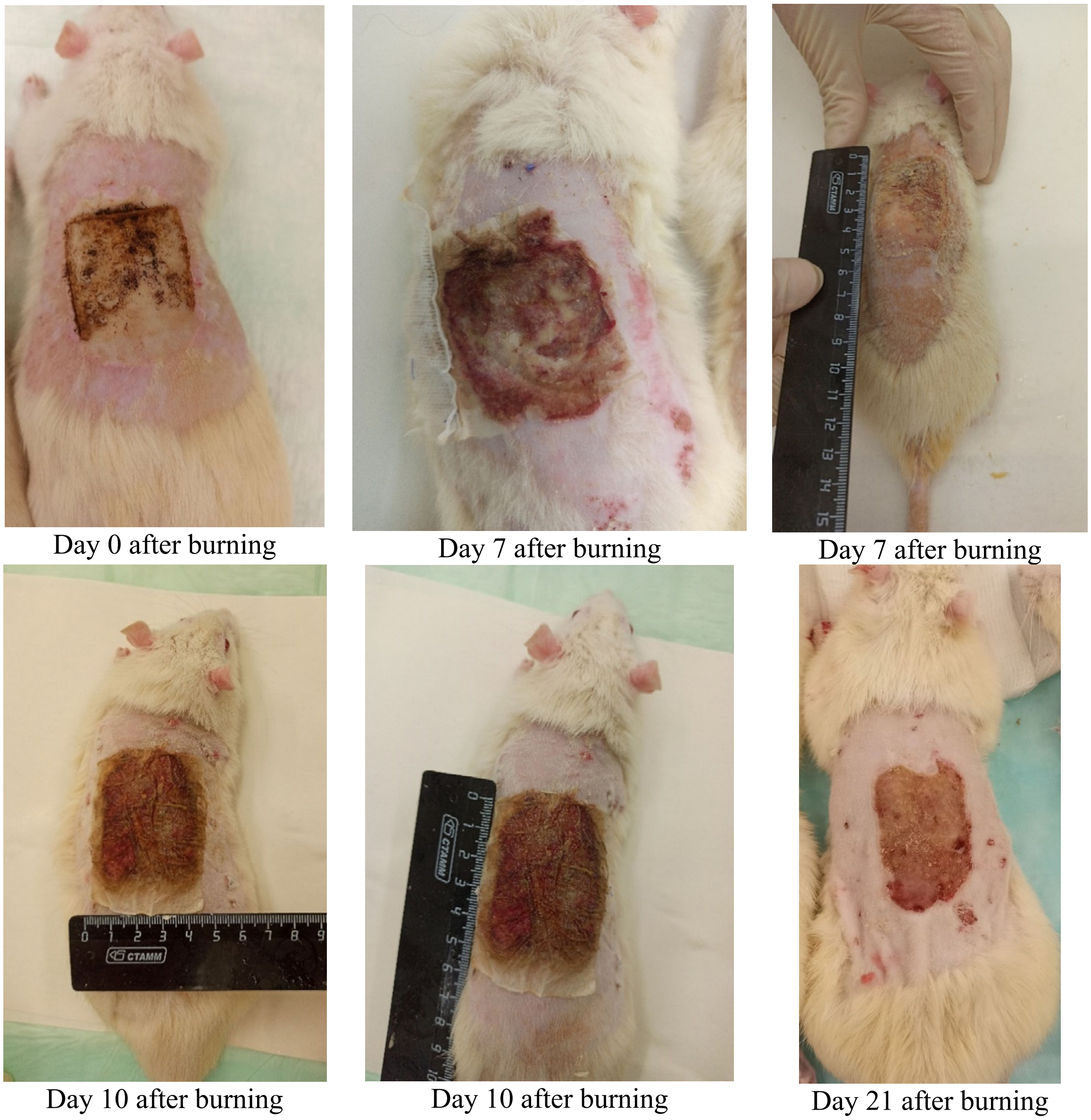

Figure S9. Treatment by BC-ZnO NPs-DBP composite.

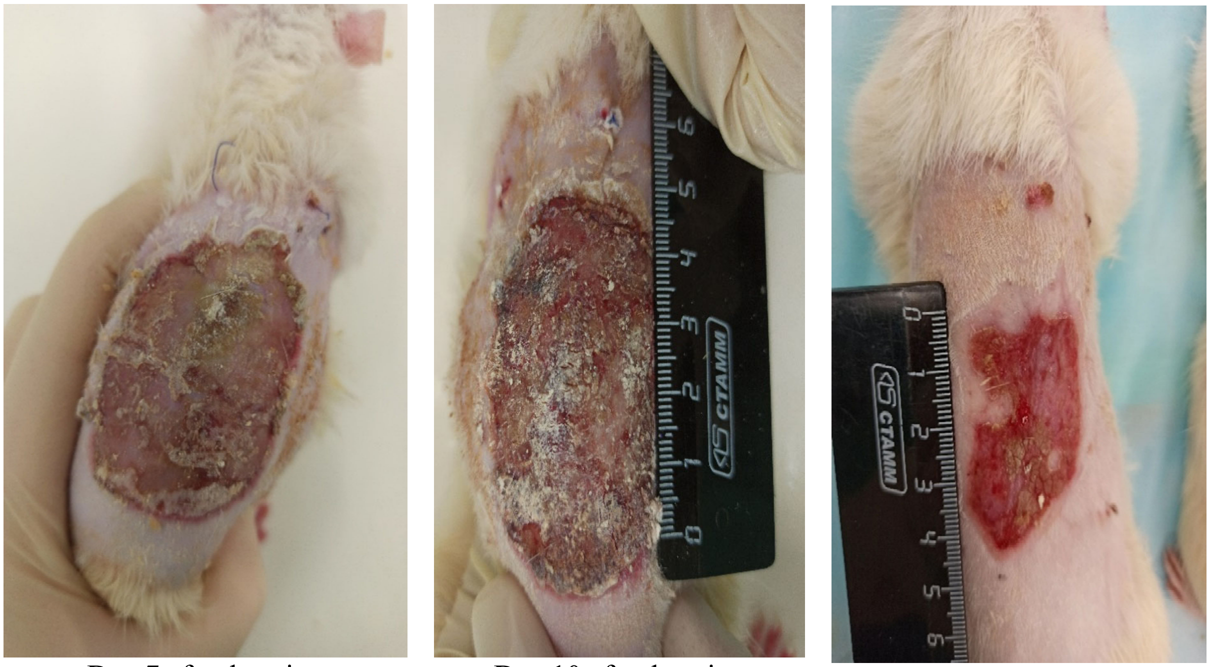

Day 7 after burning Day 10 after burning Day 21 after burning

Figure S10. Treatment by ZnO NPs-BDP-B oleogel.

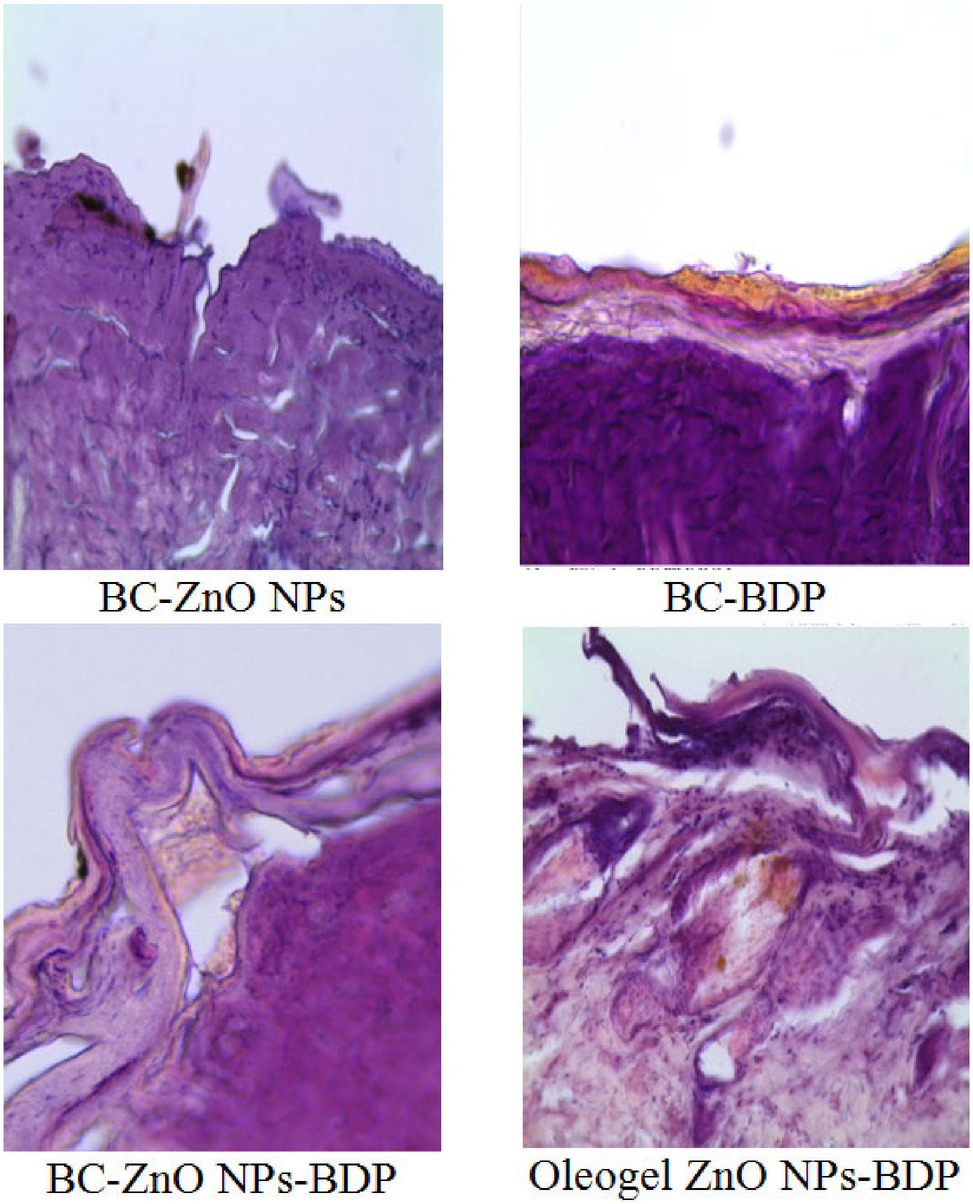

Figure S11. Tissue state by morphological and histological examination (3 Day, hematoxylin-eosin, ×600).

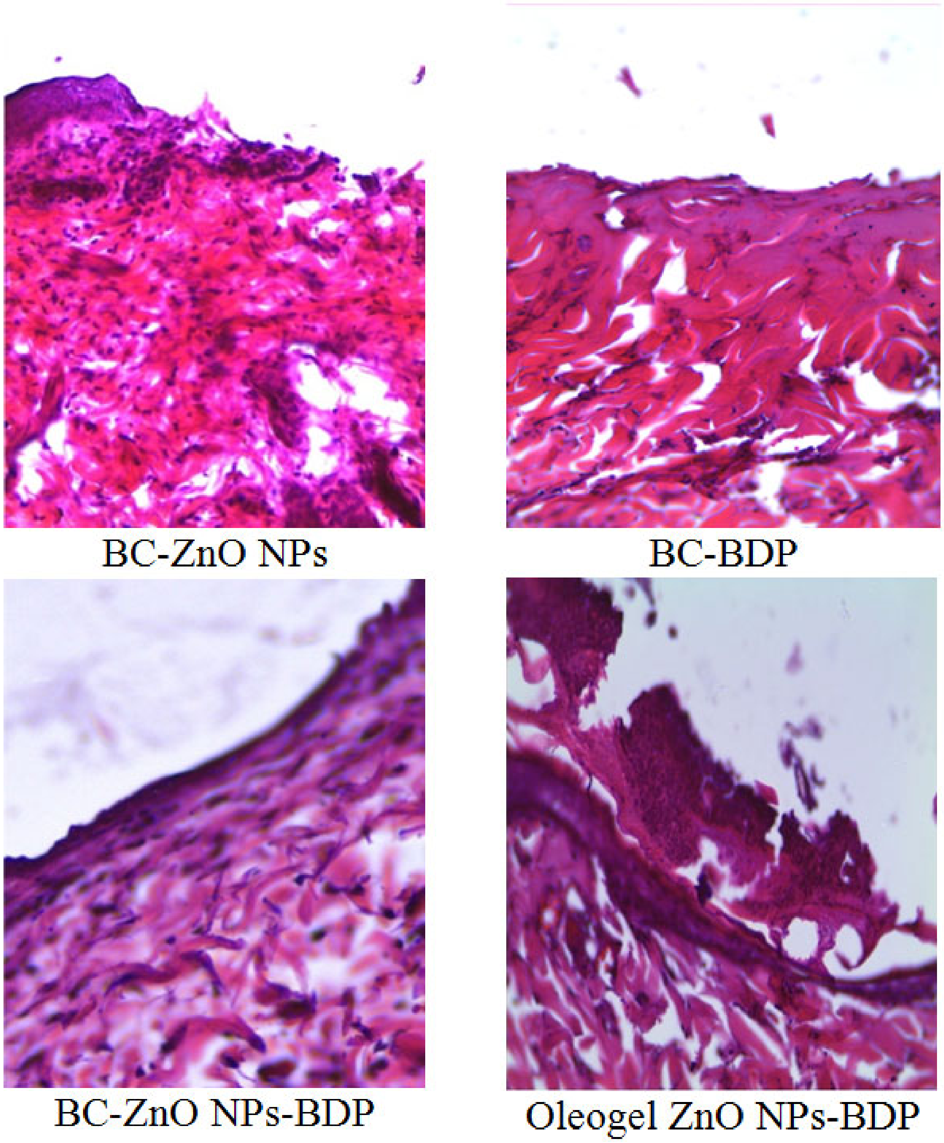

Figure S12. Tissue state by morphological and histological examination (7 Day, hematoxylin-eosin, ×600).

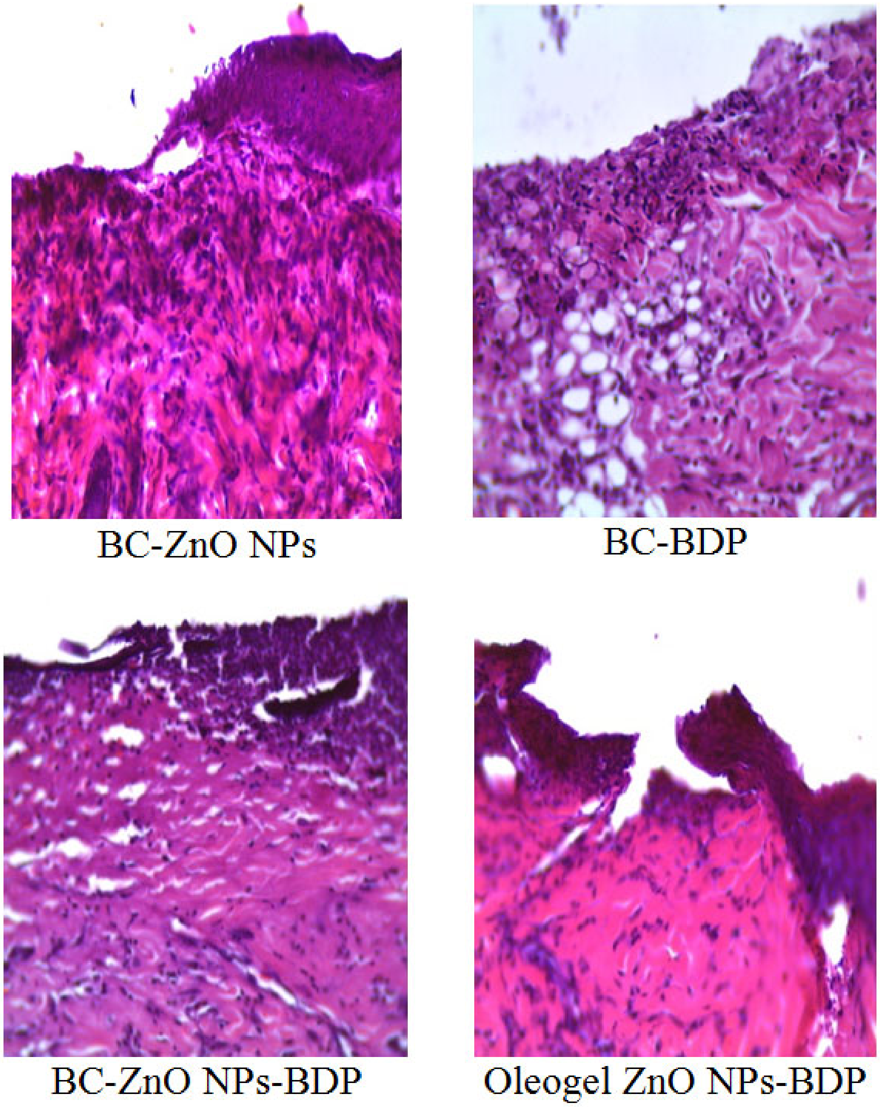

Figure S13. Tissue state by morphological and histological examination (10 Day, hematoxylin-eosin, ×600).

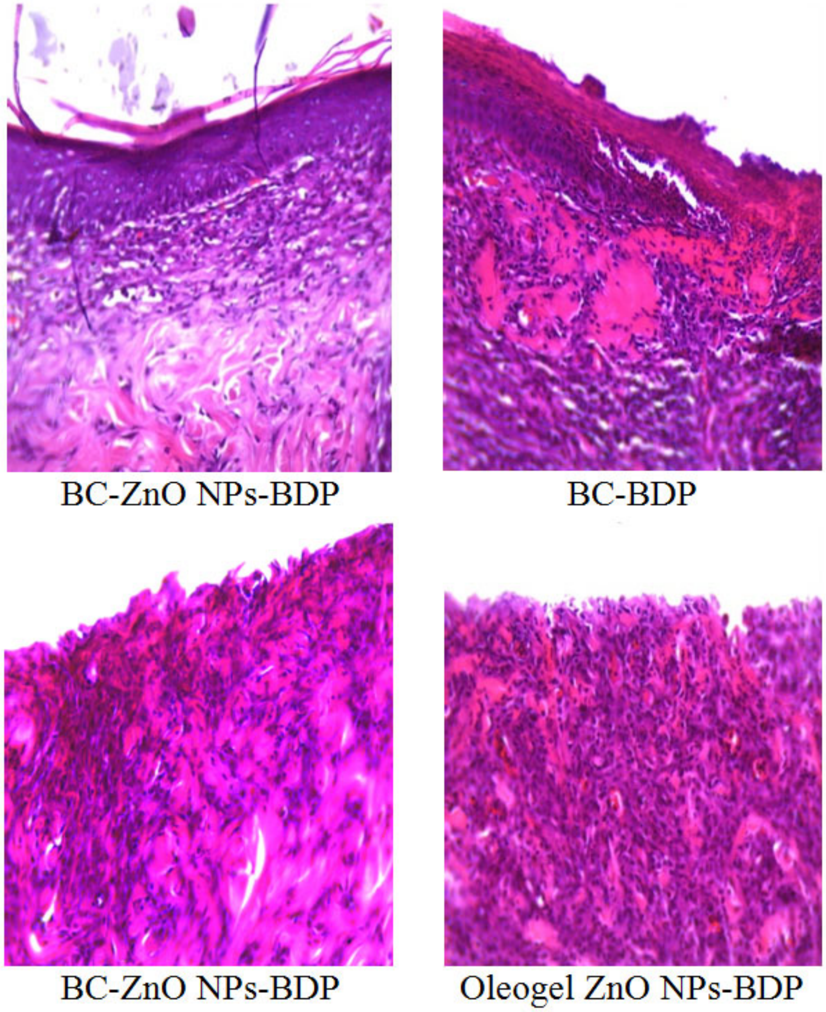

Figure S14. Tissue state by morphological and histological examination (21 Day, hematoxylin-eosin, ×600).

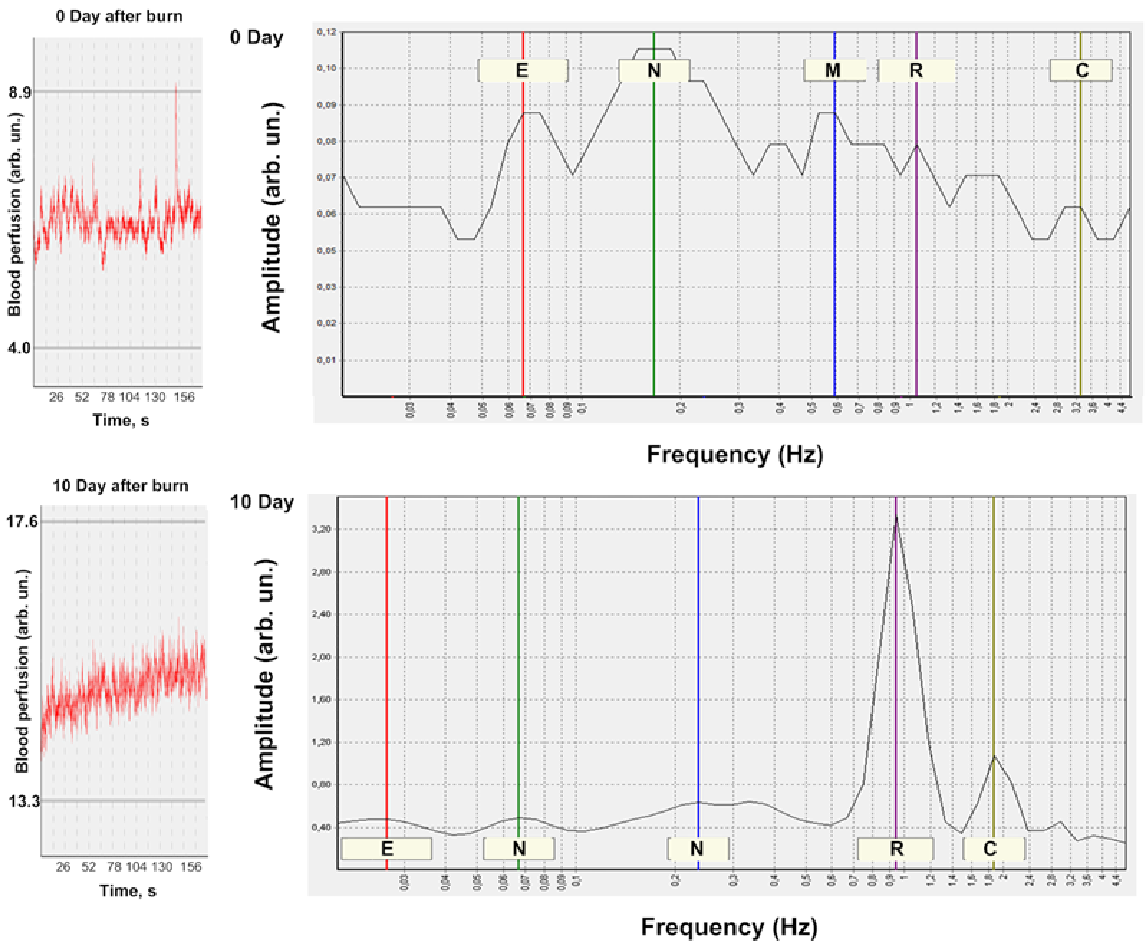

Figure S15. Typical LDF grams and the corresponding wavelet spectra obtained immediately after the burning (0 day) and on day 10 after burning on the wound of rats. E – endothelial, N – neurogenic, M – myogenic, R – respiratoty and C – cardiac oscillations.
